# Motivators and barriers to the uptake of digital health platforms for family planning services in Lagos, Nigeria: A mixed-methods study

**DOI:** 10.1177/20552076251349624

**Published:** 2025-06-09

**Authors:** Mohammed M. Alhaji, Muhammad R. Balarabe, David Atama, Arizechukwu Okafor, Doluwamu Solana, Francis Meyo, Chinedu Joseph Okoye, Uchenna Okafor, Babatunde Oyelana, Jennifer Anyanti

**Affiliations:** 1 Busara Center for Behavioral Economics, Abuja, Nigeria; 2 Busara Center for Behavioral Economics, Nairobi, Kenya; 3 575337 Society for Family Health, Abuja, Nigeria

**Keywords:** Family planning, contraceptives, digital health, e-Pharmacy, telemedicine, technology adoption, Nigeria, mixed-methods study

## Abstract

**Background:**

Inadequate access to family planning (FP) services drives low contraceptive uptake in Nigeria. Digital platforms such as e-pharmacy and telemedicine offer potential to improve access, privacy, and convenience. This study examines factors influencing provider and user adoption of digital FP services in Lagos.

**Methods:**

We used an exploratory sequential mixed-methods design. Interviews (*n =* 79) with FP providers, and current and potential users informed a survey (*n =* 1485) of digital FP platform users. Thematic analysis was applied to qualitative data; quantitative data were analyzed using descriptive, bivariate, and logistic regression methods.

**Results:**

Participants comprised online (FP and non-FP) and brick-and-mortar (FP and non-FP) users. Awareness e-pharmacies for FP was high (78%), yet usage was limited (24%). Similarly, telemedicine awareness was very low (30%), and usage was lower (6%) compared with e-pharmacy. Online platform use for FP (e-Pharmacy/telemedicine) was significantly associated (*p < *.01) with age, gender, education level, marital status and higher education. Providers identified key barriers including regulatory uncertainty, technological challenges, and concerns about privacy and quality assurance. Logistic regression suggested higher odds of e-pharmacy use among respondents aged 18–26 Respondents aged 27–35 had significantly lower odds of e-pharmacy use compared to those aged 18–26 (adjusted odds ratios (AORs) = 0.33; 95% CI: 0.22–0.48, *p < *.001), while post-secondary education predicted higher telemedicine use (AOR = 6.54; 95% CI: 2.34–18.31, *p < *.001).

**Conclusions:**

E-pharmacy and telemedicine can ease FP access, especially for younger, educated, high-income users. However, effective adoption requires addressing demand-side user barriers and supply-side provider concerns.

## Introduction

Inadequate access to family planning (FP) services remains a major driver of low uptake in many low- and middle-income countries (LMIC), including Nigeria.^1–3^ This in turn contributes to persistent levels of unmet need and adverse health and socio-economic outcomes, especially among women and adolescent girls.^4^ Despite continued efforts to expand FP access in Nigeria, many women and families still encounter barriers, and contraceptive use remains low. Only 15% of married women aged 15–49 use modern methods of contraception, while unmet need for FP remains high at 21% among currently married women and 36% among sexually active unmarried women in Nigeria.^5^ Challenges in accessing FP can lead to unintended pregnancies, which increase the risk of unsafe abortions and maternal death.^6,7^

Common barriers to contraceptive uptake in Nigeria and similar settings cut across many areas, from economic challenges and structural issues to gaps in information, clinical limitations, and sociocultural pressures.^8,9^ High out-of-pocket costs, limited availability and accessibility of services, lack of awareness and widespread misinformation, fear of side effects, social stigma and negative community perceptions have all been widely reported.^2,10–14^

Yet, digital health platforms are opening new avenues to tackle these long-standing barriers. They offer discreet, accessible, and user-centered options for accessing information, receiving counselling, and obtaining contraceptive services. By expanding the reach of services, correcting misinformation through accurate health messaging, easing financial and geographic barriers, and enhancing user privacy, digital innovations could make FP services more equitable and accessible.

With the rapid growth in internet access and mobile phone use in Nigeria (from approximately 56 million users in 2018 to over 90 million in 2023),^15^ the country's technology environment presents an opportunity to leverage digital health solutions to overcome traditional FP barriers. E-pharmacies and telemedicine are two promising approaches. E-pharmacies offer online access to medications, consultations, and delivery, including contraceptives. They offer users greater privacy and convenience, potentially mitigating the stigma or confidentiality concerns associated with obtaining FP services in person. Telemedicine refers to remote healthcare consultations conducted via the internet. This approach can help alleviate distance barriers and reduce clinic waiting times for FP consultations.

Evidence from Nigeria and other LMIC suggests that digital interventions can indeed improve FP outcomes, especially in underserved settings. Randomized trials in Kenya and India have shown that delivering FP content via mobile phones can improve contraceptive uptake and healthcare-seeking behaviors among women of reproductive age.^16,17^ In Nigeria, an interactive voice response intervention implemented in Kaduna led to increased contraceptive ideation and uptake of modern methods,^18^ while mobile messaging in another context was found to enhance spousal communication and joint fertility decision-making^19^ Beyond specific interventions, systematic reviews consistently demonstrate that mobile health (mHealth) strategies, such as SMS reminders, mobile applications and helplines positively influence contraceptive initiation and continuation.^20,21^ Much of this evidence is centred around informational and behavioral interventions rather than digital transactional platforms. However, emerging Nigerian studies on e-pharmacy and telemedicine have identified convenience, privacy, and user autonomy as key motivators for adoption, while concerns around provider legitimacy, digital literacy, and regulatory clarity continue to pose barriers.^22–24^ These findings underscore the potential of digital health strategies in healthcare access.

Despite these encouraging findings, the adoption of available digital health platforms is not often guaranteed. Technology adoption theories provide some insights. The Technology Acceptance Model (TAM), for example, posits that perceived usefulness and ease of use are key drivers of technology adoption. If users do not perceive a clear benefit or find a digital platform easy to use, they may be reluctant to adopt it, resulting in low uptake.^29^ Similarly, the Diffusion of Innovations (DOI) theory highlights the roles of innovators and early adopters, and emphasizes characteristics like relative advantage, compatibility, and complexity that affect how a new technology spreads.^30^ In line with this theory, digital FP platforms in Nigeria appear to be in an early adoption phase. They are used mostly by younger, tech-savvy individuals and have not yet become mainstream.^14^ The Health Belief Model (HBM), in addition, focuses on how perceived severity, susceptibility, benefits, and barriers shape health behaviors.^31,32^ Using the HBM, a woman's choice to use e-pharmacy for FP may hinge on her perceived need and benefits of the service, balanced against concerns like trust and cost.

Despite the promise of digital platforms and the guidance of these theoretical frameworks, the use of digital technologies for FP in Nigeria remains under-researched. Few studies have examined these platforms in relation to FP service delivery. Critical knowledge gaps include understanding the motivators and barriers to adoption at individual, household, and system levels; examining how socio-demographic factors influence uptake; and further clarifying alignments in provider and user perspectives. This study aims to fill these gaps by investigating the motivators and barriers to uptake of digital FP platforms, specifically e-pharmacy and telemedicine, in Lagos, Nigeria. Findings from this study aim to inform interventions and policies that enhance the use of digital health for FP in Nigeria, supporting FP2030 goals and advancing progress toward SDG Targets 3.7 and 5.6 on reproductive health access.

## Methods

### Study design and setting

We employed an exploratory sequential mixed-methods design, comprising an initial qualitative phase followed by a survey. The qualitative component of the study employed an exploratory qualitative design informed by phenomenological principles (exploration of participants’ lived experiences with digital platforms for FP and thematic analysis. First, we undertook in-depth interviews (IDIs) to gather rich, contextual insights from both end-users and providers of digital FP services. Building on these findings, we designed a cross-sectional survey to measure the extent to which those insights hold in a broader population.

The study was carried out in Lagos, Nigeria, with a focus on urban areas. Lagos was chosen due to its diverse population, robust digital infrastructure, and active e-health environment. Data collection took place between September and December 2023. At the time of the study, Lagos had relatively high internet coverage and a mix of public and private health facilities, including emerging digital health services.^33^

### Study population

The study population comprised participants from both the demand and supply sides of digital health engagement for FP services. On the demand side, the study population included adults aged 18 years and older who had either used or had the potential to use digital health platforms for FP. Participants were classified into four mutually exclusive groups based on reported use of digital health platforms and FP services. For the qualitative phase, broad user types guided participant selection, while for the quantitative survey categorization was performed post-hoc during analysis. This classification enabled systematic comparisons across different patterns of service use.

“Online FP Users” were individuals who had recently obtained FP products or services through digital platforms, such as ordering contraceptives from an e-pharmacy or consulting via telemedicine. “Online Non-FP Users” referred to individuals who used digital health services for other medical needs but had not accessed FP services online, offering insights into barriers to digital FP uptake even among tech-savvy users. “Brick-n-Mortar” (BnM) FP Users were individuals who accessed FP services exclusively through traditional in-person channels like clinics, hospitals, or physical pharmacies. Finally, “BnM Non-FP Users” were those not currently using any FP methods, either online or offline, providing a reference group to explore baseline awareness and attitudes toward FP.

On the supply side, the study targeted digital FP service providers, specifically licensed e-pharmacists and telemedicine physicians operating in Lagos. Eligible providers were required to have a minimum of three years of professional experience and active involvement in delivering FP services through digital channels, ensuring the inclusion of perspectives from experienced practitioners familiar with both healthcare delivery and digital service models.

### Sample size

The qualitative sample size was determined based on the principle of data saturation, whereby interviews were conducted until no new themes emerged. A total of 79 IDIs were completed, comprising 49 end-users and 30 digital health service providers (14 e-pharmacists and 16 telemedicine physicians). This sample size was deemed sufficient to capture a diverse range of experiences, perceptions, and barriers across user categories, while ensuring thematic saturation and depth of insight.

The quantitative sample size was calculated using standard formulae for estimating proportions in cross-sectional surveys (*n =* *z*^2^ *pq*/*d*^2^).^30^ Assuming a 50% prevalence (*p*) (to ensure maximum variability), a 95% confidence level (*z*), and a 5% margin of error (*d*), the minimum required sample size was estimated at 384 respondents. To account for potential non-response and to enable subgroup analyses across user categories, the final sample size was expanded to 1485 participants, yielding a sample size with over 90% statistical power to detect modest differences between key groups at a 95% confidence level.

### Sampling technique

For the qualitative phase, we employed purposive sampling to recruit a diverse yet relevant sample of participants. E-pharmacy and telemedicine providers were identified through professional directories, online platforms, and referrals, while end-users and potential users were recruited via partnerships with digital health services, engagement at urban clinics and pharmacies, and snowball sampling. Eligible participants were adults aged 18 years or older, residing in Lagos for at least one year, proficient in English, and willing to provide informed consent.

A multistage sampling approach was adopted for the survey. First, five urban Local Government Areas (LGAs) in Lagos State were purposively selected based on high internet connectivity and the presence of ongoing FP interventions. These criteria aligned with the study's objective to explore digital FP use. In each LGA, two wards were randomly selected from official listings to ensure geographic diversity. Within each ward, households were randomly selected and one eligible adult (aged 18–49 years with mobile phone access) per household enrolled for the study. Respondents were further stratified by sex using a 4:1 female-to-male quota to ensure strong female representation while capturing male perspectives relevant to household FP decisions.

### Data collection instruments and procedure

For the qualitative phase, two semi-structured interview guides (one each for end-user and provider) were developed based on the literature and research questions. The interview guides explored participants’ awareness of digital health platforms, experiences and perceptions of e-pharmacy and telemedicine for FP, use of digital platforms for FP services, perceived motivators and barriers, and suggestions for improving services. Interview guides were reviewed by stakeholders, piloted among a small group (*n =* 13), and refined for clarity.

Quantitative data were collected using a structured questionnaire developed in English, informed by insights from the qualitative phase and existing surveys on digital health and FP in sub-Saharan Africa. The questionnaire covered socio-demographic characteristics, FP knowledge and practices and engagement with digital health platforms. Key sections explored participants’ age, marital status, education, income, contraceptive use history, sources of FP services, awareness and use of e-pharmacies and telemedicine platforms alongside motivators and barriers encountered. The instrument was reviewed by FP experts to ensure relevance and clarity and was piloted among a small group of ten individuals, resulting in minor refinements.

All interviewers received a one-day training before fieldwork commenced. The training covered the study protocol and all study data collection tools. It also included ethical considerations in studies involving human participants and techniques for effective probing and rapport-building. For the qualitative aspect, emphasis was placed on maintaining neutrality and minimizing interviewer bias. They were further sensitized to how their worldview and positionality could influence data collection processes and participant engagement. This helped foster reflexivity throughout the study. Continuous oversight and regular debriefing sessions enhanced consistency and quality throughout the interview process.

Four interviewers (MRB, AO, DS, and CJO—all with prior qualitative research experience) conducted IDIs in English, either in private settings in participants’ home, office or via secure video calls, based on participants’ preferences. Interviews lasted 30–60 minutes and were audio-recorded with consent. In addition, field notes were taken during sessions. No prior relationships existed between interviewers and participants. All recordings were transcribed verbatim, anonymized, and cross-verified for accuracy by a second team member.

Quantitative data collection was primarily conducted in person using electronic tablets equipped with SurveyCTO software for real-time data capture. In some instances, participants completed the survey independently through a secure online link. Field supervisors monitored data collection daily, identifying and addressing fieldwork challenges and data entry inconsistencies. These quality assurance measures helped ensure the integrity and reliability of the final dataset.

### Data analysis

Qualitative data were analyzed, using a thematic analysis approach, was conducted with NVivo 12 software. An initial coding framework, informed by the research questions and interview guide, focused on awareness and utilization of e-pharmacy and telemedicine for FP services and associated motivators and challenges. Sub-themes such as trust issues, technical barriers, cost concerns, privacy issues, and lack of awareness were inductively identified during transcript review. Two researchers (MRB, DS) independently coded a subset of transcripts to ensure intercoder reliability, with discrepancies resolved through consensus. The finalized codebook guided further coding and thematic matrix development, enabling comparisons across user groups. Qualitative findings were used to complement quantitative results, with illustrative quotes selected to highlight key themes and participant perspectives.

Quantitative data were analyzed using IBM SPSS (Version 29.0). Descriptive statistics summarized participant characteristics and key indicators by user categories (Online FP, Online non-FP, BnM FP, and BnM non-FP). Bivariate analyses, using chi-square test, assessed associations between participants’ socio-demographic characteristics and digital platform use for FP, with the level of significance set at *p < *.05. Multivariate logistic regression (MLR) models were then constructed to identify independent predictors of use if online platform for FP. Factors included in the models were age group, gender, education level, marital status, employment status, and income. Adjusted odds ratios (AORs) with 95% confidence intervals were calculated for each predictor. Smartphone ownership was excluded from the e-pharmacy model due to near-universal access among users.

### Data integration

Findings from this mixed-methods study were presented and interpreted in an integrated manner. Quantitative and qualitative results were compared to develop a cohesive understanding, with the findings reported in a unified narrative and illustrative quotes used to enrich the interpretation.

### Ethical considerations

This study was approved by the Nigerian National Health Research Ethics Committee (NHREC) (NHREC/01/01/2007-23/05/2023), ensuring that all procedures complied with ethical standards for research involving human participants. All participants gave informed consent. Written consent was obtained for in-person interviews; verbal consent was recorded for virtual ones. Participation was voluntary, confidential, and could be withdrawn at any time. Data materials were securely stored, and electronic devices were password protected.

## Results

This study employed a mixed-methods approach, combining quantitative survey findings and qualitative interview insights to provide a comprehensive understanding of digital FP platform uptake. Thematic analysis of the qualitative interviews generated four major themes: motivators for use of digital platforms, demand-side barriers to uptake, supply-side barriers to uptake, and trust and credibility concerns. Quantitative findings are reported alongside supporting qualitative narratives to highlight points of possible convergence, complementarity, or divergence.

### Participant distribution and digital platform use by study phases

A total of 79 individuals (49 end-users and 30 service providers) took part in the qualitative interviews while 1485 participants were enrolled in the survey. Table 1 presents the distribution of participants across digital health user categories and study phases. Among survey respondents, approximately 46% reported using a digital health platform with 24% specifically using it for FP services and 22% for other health-related needs. The remaining participants accessed services exclusively through traditional, physical facilities (29.3% for FP and 24.6% for other services. The qualitative sample reflected all user categories, with a slight over-representation of digital platform users to support richer insights into their experiences and use patterns.

**Table 1.** Distribution of respondents across study phases and user categories.

Categories	Respondents			
	Qualitative phase		Quantitative phase	
	*n*	%	*n*	%
*Demand-side (end-users of health services)*				
Online FP Users	18	36.7	358	24.1
Online Non-FP Users	16	32.7	326	22.0
Brick & Mortar (BnM) FP Users	10	20.4	435	29.3
Brick & Mortar (BnM) Non-FP Users	5	10.2	366	24.6
Total	49	100	1485	100.0
*Supply-side (digital health providers)*			-	
e-Pharmacists	14	46.7	-	
Telemedicine Providers	16	53.3	-	
Total	30	100.0	-	

### Sociodemographic profile of study participants

In the qualitative interviews, 80% of the participants were women, aged between 27 and 35 years, married, had post-secondary education, and reported earning less than NGN 200,000. Among the 30 providers, 60% were female and 47% were aged 18–26 years. Table 2 presents the socio-demographic characteristics of all the survey participants. The sample was predominantly female (82%), with Online FP users accounting for the majority (90%) of participant within the group. The median age was 29 years (IQR: 24–34), and 71% were aged 18–26 years, and accounted for the majority among all user categories. BnM FP users had a slightly older age profile, with 28% and 12% aged 27–35 and 36–45, respectively.

**Table 2.** Socio-demographic profile of survey respondents by user category (*n =* 1485).

Variable	Brick and mortar FP users	Online FP users	Brick and mortar non-FP users	Online non-FP users	Total
	*n* (%)	*n* (%)	*n* (%)	*n* (%)	
*Gender*					
Female	348 (80)	322 (90)	285 (78)	267 (82)	1222 (82.2)
Male	87 (20)	36 (10)	81 (22)	59 (18)	263 (21.8)
*Age group (years)*					
18–26	261 (60)	254 (71)	282 (77)	254 (78)	1051 (70.8)
27–35	122 (28)	72 (20)	47 (13)	46 (14)	287 (19.3)
36–45	52 (12)	32 (9)	37 (10)	26 (8)	147 (9.9)
*Marital status* ^a^					
Cohabiting	2 (<1)	2 (<1)	5 (<1)	1 (<1)	10 (<1)
Married	304 (21)	169 (11)	157 (11)	101 (7)	731 (50.2)
In-relationship	77 (5)	83 (6)	84 (6)	85 (6)	329 (22.6)
Separated/divorced/widowed	5 (<1)	8(<1)	6 (<1)	1 (<1)	20 (1.4)
Single	44 (3)	69 (5)	114 (8)	138 (9)	365 (25.1)
*Education*					
None	3(0)	0 (0)	0 (0)	0 (0)	3 (0.2)
Primary	8(2)	0 (0)	8(2)	0 (0)	16 (1.1)
Secondary	142 (33)	28 (8)	152 (42)	34 (11)	356 (24.0)
Post-secondary	282 (65)	330 (92)	206(56)	292 (89)	1110 (74.7)
*Employment*					
Employed	113 (26)	79 (22)	102 (28)	81 (25)	375 (25.3)
Business/self-Employed	261 (60)	200 (56)	208 (57)	179 (55)	848 (57.1)
Unemployed	39 (9)	21 (6)	18 (5)	23 (7)	101 (6.8)
Student	22 (5)	61 (17)	36 (11)	42 (13)	161 (10.8)
*Income level (NGN)*					
Below 100,000	278 (64)	251 (70)	124 (34)	150 (46)	803 (54.1)
100,000–200,000	109 (25)	82 (23)	176 (48)	114 (35)	481 (32.4)
201,000–300,00	31 (7)	14 (4)	44 (12)	46 (14)	135 (9.1)
More than 300,000	17 (4)	11 (3)	22 (6)	16 (5)	66 (4.4)

a Non-responses are excluded (*n =* 30).

Nearly half of BnM FP users were married (49%) compared to 47% of Online FP users and 26% of Online non-FP users. The majority, 75%, had post-secondary education, with 92% of Online FP users and 89% of online non-FP users having attained this level of education. Business or self-employment was the most common employment category overall (57%) and more than half across all user categories. Over half of respondents (54%) reported monthly incomes below ₦100,000. Higher income levels (≥₦200,000) were more common among online users. Almost all participants (96%) reported ownership of a smartphone, and all participants had access to a mobile phone.

### Awareness and use of e-pharmacy for family planning products

**Figure 1.**
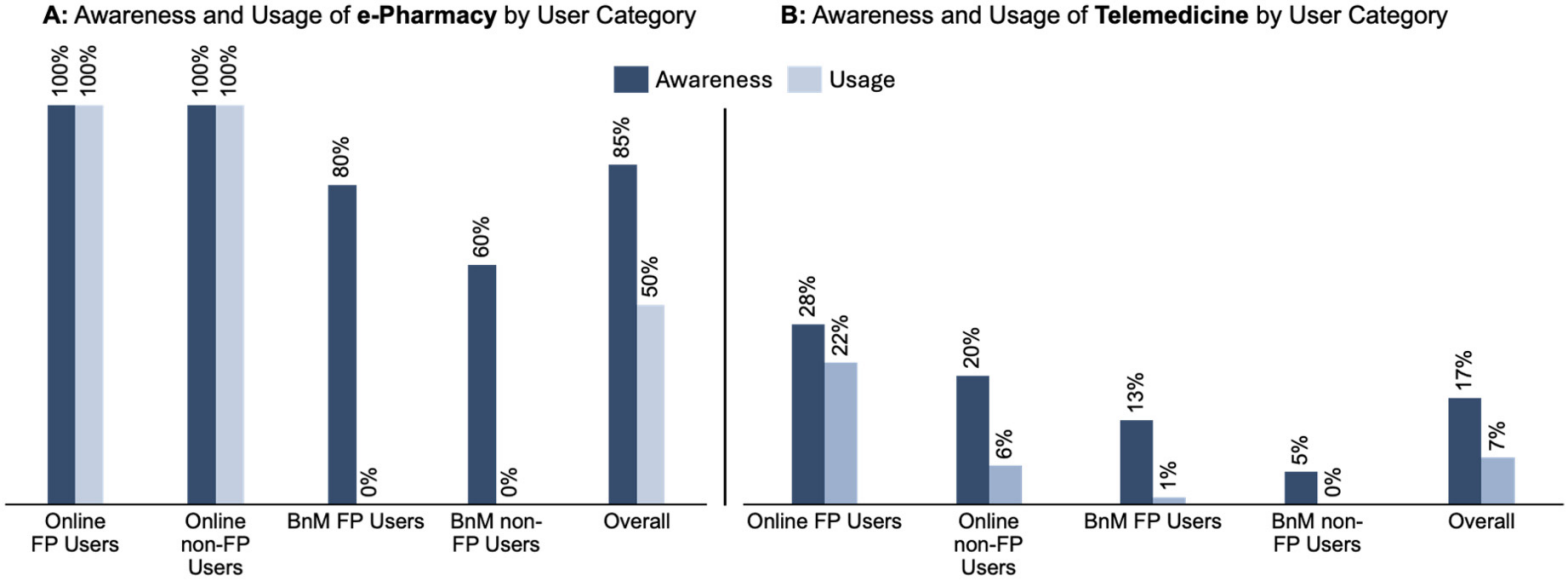
Awareness and the use of e-pharmacy and telemedicine platforms for family planning among survey respondents.

Overall, 78% of respondents were aware of e-pharmacy services. However, only 24% had ever used these platforms for the purpose of FP services (Figure 1A). Awareness of e-pharmacy was nearly universal among Online FP and Online non-FP users (100% in both groups), while comparatively slightly lower among BnM FP users (80%) and BnM non-FP users (60%). Approximately, only 10% of BnM FP users reported having tried online ordering for contraceptives.

Among those who had used e-pharmacy services for FP, oral contraceptive pills (63%), male condoms (42%), and emergency contraceptives (36%) were the most frequently purchased products. Long-acting reversible contraceptives such as self-injectables, for example, DMPA-SC (9%) and implants (17%) were less frequently obtained online.

Bivariate analysis (Table 3) showed that e-pharmacy use was significantly associated with age (particularly 18–26 years), gender, education level, employment, and marital status (*p < *.001 for most variables). Multivariate logistic regression (Table 4) showed that respondents aged 27–35 years (AOR = 0.55; 95% CI: 0.35–0.85, *p = *.002) and 36–45 years (AOR = 0.45; 95% CI: 0.28–0.75, *p = *.001) had significantly lower odds of using online FP services compared to those aged 18–26. Male respondents (AOR = 0.46; 95% CI: 0.30–0.70, *p = *.001) and those with secondary education or less (AOR = 0.25; 95% CI: 0.15–0.42, *p < *.001) were also significantly less likely to use online FP platforms. Being married was associated with reduced odds of use (AOR = 0.50; 95% CI: 0.32–0.78, *p = *.001), while other marital categories did not show significant associations.

**Table 3.** Bivariate comparison of socio-demographic characteristics by use of online FP versus brick-and-mortar family planning services (*n =* 793).

Variable	Brick and mortar FP users	Online FP users	χ²	*p*
	*n* (%)	*n* (%)		
*Gender*			14.07	<.001
Female	348 (80)	322 (90)		
Male	87 (20)	36 (10)		
*Age group (years)*			10.4	.0056
18–26	261 (60)	254 (71)		
27–35	122 (28)	72 (20)		
36–45	52 (12)	32 (9)		
*Marital status*			32.2	<.001
Married	305 (21)	187 (11)		<.001
In-relationship/cohabiting	81 (5)	91 (6)		.390
Separated/divorced/widowed	5 (<1)	9(<1)		.310
Single	44 (3)	71 (5)		.015
*Education*			81.9	<.001
Secondary or less	153(35)	28 (8)		
Post-secondary	282 (65)	330 (92)		
*Employment*			14.2	.003
Employed	113 (26)	99 (22)		.210
Business/self-employed	261 (60)	203 (56)		.340
Unemployed	39 (9)	17 (6)		.080
Student	22 (5)	39 (17)		<.001
*Income level (NGN)*			5.5	.140
Below 100,000	278 (64)	251 (70)		.070
100,000–200,000	109 (25)	82 (23)		.540
201,000–300,00	31 (7)	14 (4)		.090
More than 300,000	17 (4)	11 (3)		.420

**Table 4.** Multivariate logistic regression of factors associated with the use of online platforms for family planning services (*N =* 793).^a^

Variable	AOR	95% CI	*p*
*Age group (Ref: 18–26)*			
27–35	0.550	(0.350–0.850)	.002
36–45	0.450	(0.280–0.750)	.001
*Sex (Ref: Female)*			
Male	0.460	(0.300–0.700)	.001
*Education (Ref: post-secondary)*			
Secondary or less	0.250	(0.150–0.420)	<.001
*Marital status (Ref: single)*			
Married	0.500	(0.320–0.780)	.001
In-relationship/cohabiting	0.800	(0.550–1.200)	.300
Separated/divorced/widowed	1.500	(0.900–2.500)	.200
*Income level (Ref: ₦100,000–200,000)*			
>₦200,000	0.900	(0.550–1.450)	.500
Below ₦100,000	0.700	(0.480–1.000)	.030
None	0.650	(0.380–1.100)	.020
*Employment status (Ref: Employed)*			
Business/self-employed	0.900	(0.600–1.350)	.400
Unemployed	0.400	(0.200–0.800)	.005
Student	2.100	(1.300–3.400)	.010

a A potential limitation is untested multicollinearity, especially among income, and employment, which may inflate standard errors and affect estimate stability. Future analysis should assess this using diagnostic tools like variance inflation factors.

Qualitative interviews similarly revealed that awareness of e-pharmacy services was relatively widespread among participants, though their understanding of its application for FP varied. Many respondents had encountered online pharmacies through advertisements, social media, or personal networks.

Yes, I have used one of these online pharmacy platforms…I actually got to know them through a friend. (25-year Female FP user participant)

Several participants had used e-pharmacy platforms to purchase contraceptive pills, condoms, or emergency contraceptives, particularly those familiar with digital transactions. However, some participants reported limited or incorrect understanding, with a few assuming that online pharmacies were only suited for common medications or non-prescription items.

I do log in to check what they have in store, but I've not purchased from them… when I need some drugs, and I don't know the actual drug to go for… I just write the name of the drug, and they tell me what it is used for, the cost, and usage. (Female, 38 years old)

### Awareness and use of telemedicine for family planning services

Approximately 30% of respondents reported familiarity with the concept of remote consultations, with lower levels of awareness specific to FP services (Figure 1B). Among user categories, BnM non-FP users exhibited the lowest awareness (5%), while Online FP users had the highest (28%). Overall, 6% of respondents had utilized telemedicine services for FP, and approximately 10% had used telemedicine for any healthcare need. Telemedicine users were predominantly drawn from the Online FP user group, with very low uptake (1%) among BnM users.

Bivariate analysis (Table 3) showed that higher education, higher income, and marital status were significantly associated with telemedicine use (*p < *.05).

Awareness of telemedicine platforms was less common among qualitative interview participants compared to e-pharmacy. Very few of the participants had heard of or used digital consultations for FP services.

…telemedicine is like a conversation… when you are trying to get across to a doctor online…. I haven't heard of any [telemedicine] providers in Lagos. (Female, 25 years old)

Among those who had used telemedicine, experiences included virtual discussions with doctors regarding contraceptive side effects, prescription renewals, or method switching. Most users of telemedicine platforms described the process as straightforward and appreciated the flexibility of remote access.

I use it for guidance and health counselling… mainly via a smartphone, as apps are better on phone. (Male, 28 years old)

### Motivators for use of digital health platforms

Among participants who used digital platforms for FP, the principal motivators included the following:

#### Convenience and time savings

**Figure 2.**
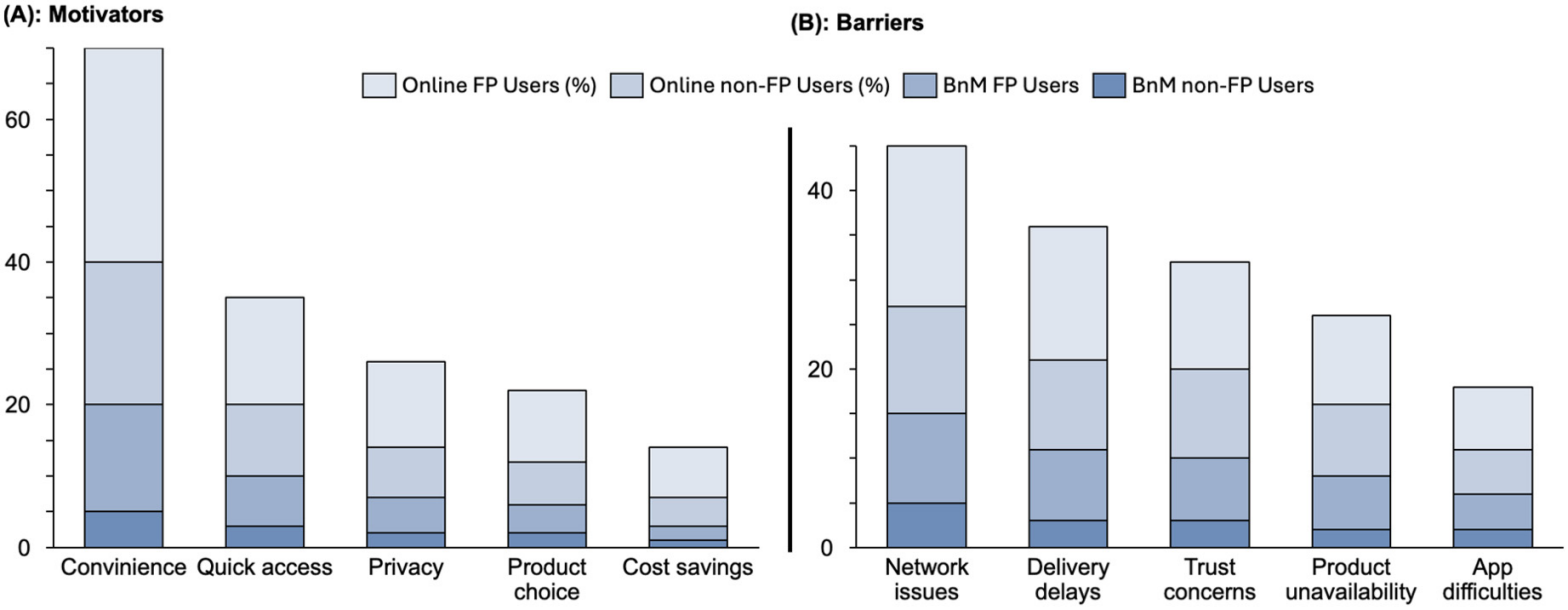
Key motivators and barriers to the use of digital platforms for family planning.

Figure 2 indicates that 72% of digital users reported convenience of being able to order products or consult providers online as a major motivator. Qualitative findings also suggested that reduced travel and time burden associated with online services were major incentives for adoption.

I can just place an order during my lunch break and get my pills delivered, instead of spending half a day at the hospital. (Female, 29 years, FP User)

#### Quick access to services

This was reported by 35% of survey respondents. Furthermore, qualitative interviews reveal that participants particularly mentioned quick access in relation to swift consultations or access to scarce products especially by users navigating congestion in Lagos to reach physical clinics.

Online platforms are available 24/7 and I could get advice for minor questions promptly. (Female, 26 years, FP User)

#### Cost savings

Although mentioned by only 13% of survey participants, qualitative interviews revealed that some users acknowledged that e-pharmacies occasionally offer discounts.

I got a bulk discount on a 3-month supply of pills online, which my local pharmacy didn’t offer. (32 years, Female, FP Users)

However, other participants balanced this with the observation that delivery fees sometimes offset savings for online services.

Yes, when you ordered drugs, you still have to pay for delivery… When compared with the physical pharmacy store, there is not cost of delivery, you just walk in and get what you want. (Male, 28 years)

#### Privacy and reduced stigma

This theme emerged prominently in qualitative interviews, especially among unmarried women, indicating how digital channels help them to avoid judgmental encounters.

It comes in a package with no labels, so even my neighbors don’t know what's inside. (Female, 22-year-old respondent)

#### Greater choice of products and information

From the qualitative interviews, Online FP users highlighted and appreciated the variety and educational resources made available online.

I learned about the self-injectable contraceptive (DMPA-SC) through an app, which I wouldn’t have known to ask for otherwise. (Female, 28 years, FP User)

### Barriers to uptake of digital family planning platforms

Overall, multiple barriers were identified that constrain the broader uptake of digital health platforms for FP. These challenges were thematically classified into demand-side barriers (faced by end-users) and supply-side barriers (encountered by service providers).

### Demand-Side barriers

#### Limited awareness of e-pharmacy and telemedicine

Qualitative interviews revealed that many potential users lack awareness of the full range of FP services available through e-pharmacy and telemedicine. Some participants, while admitting their limited knowledge, expressed a desire for more information.

To be sincere, I don't know. I've not really done online pharmacy stuff, …. So, I prefer going one-on-one to the pharmacy or to the hospital. (BnM User, 43 years)

#### Trust issues

Findings from the qualitative interviews showed that some online FP users were skeptical about the authenticity of online services and the qualifications of service providers who render the services.

Like the person I'm talking to now online - how am I able to trust this person's qualification? (FP User, 26 years).

#### Preferences for in-person interactions

From the qualitative findings, older or less educated individuals showed a preference for face-to-face interactions with service providers due to a desire for personalized care, direct questioning, and greater perceived trust in physical consultations.

Most people prefer to go into the facility, especially the elderly ones. (Non-FP User, 29 years)

#### Technical challenges

Internet network issues were reported by 64% of online FP users and 58% of online non-FP users as a major challenge among survey participants. Similarly qualitative findings revealed issues including difficulties navigating digital platforms, poor network connectivity, and language barriers.

To be sincere, I've not really done online pharmacy stuff, you know… So, I prefer going one-on-one to the pharmacy or to the hospital. (BnM User, 43 years)

…One [issue] will be the lack of technical know-how, which means the app does even have an issue, but this patient does not know where to go to… the person does not know that's where they are supposed to click. (TMP, 27 years)

#### Cost and payment barriers

Cost-related factors were a significant barrier to the use of digital platforms for FP services. These included delivery fees, the absence of health insurance coverage. Participants specifically reported that rising transportation and delivery costs, exacerbated by fuel scarcity and inflation, negatively influence their willingness to purchase products online.

…especially now that there is this fuel scarcity and high price. Delivery is very, very much now [expensive]… So sometimes it discourages people from buying online. (Female 25 years, FP user)

Furthermore, concerns about the safety and reliability of digital payments were reported by participants as barriers for uptake of digital platforms, particularly among users with limited digital literacy or prior negative experiences with online transactions.

#### Cultural beliefs and myths

Misconceptions and deep-seated cultural beliefs about contraceptives were also found to shape service-seeking behavior. Participants cited fears of infertility, perceived moral decline associated with contraceptive use, and misinformation spread through community and peer networks. These beliefs contributed to hesitation or outright refusal to engage with both online and offline FP services.

### Supply-Side barriers

#### Telecommunication, connectivity other infrastructure limitations

Operational challenges related to telecommunications infrastructure were commonly reported by digital health providers as limitations that hindered consistent service delivery and user dissatisfaction. Poor network coverage and frequent internet disruptions in some areas adversely affected the quality and timeliness of online consultations. This was also reflected in the quantitative data, where 64% of online FP users and 58% of online non-FP users identified network issues as a major challenge to using digital platforms.

There might be a few issues…network problems for example if she has network issues, sometimes there might be a slight delay in attending to you [her]. (e-Pharmacist, 37 years)

#### Client verification and communication barriers

Inaccurate or incomplete user-provided contact details were noted as a frequent challenge, impeding follow-up communication and delaying service completion. Providers expressed frustration with their inability to sometimes reach their clients after initial consultations.

…the number they give to you, you might not necessarily be able to reach them. (27 years).

#### Verification and regulatory gaps

Gaps in prescription verification processes and unclear regulatory frameworks posed significant challenges to effective delivery of digital FP services. Providers reported frequent issues with invalid or outdated prescriptions, missing contact details, and attempts to bypass verification requirements. These challenges were more pronounced in the context of e-pharmacy services where sometimes users intentionally remove prescription dates. Quantitative findings revealed that 18% of online FP users and 42% of online non-FP users experienced prescription-related difficulties.

It's just the adamant ones, who want a drug given even though the prescription is old or outdated, or some will scan a prescription where they will cut off the dates you know, and they will want you to accept it. (e-Pharmacist, 35 years)

#### Clinical limitations of remote care

Service providers highlighted the inability to perform physical examinations as a critical limitation of telemedicine. The reliance on patients’ subjective reports was seen as a constraint on diagnostic accuracy and clinical decision-making. This challenge was particularly evident in contexts where users misrepresented symptoms or withheld information.

The fact that we cannot examine the patient is one of the greatest limitations in telemedicine … We can't make use of [a] stethoscope…A lot of things can go wrong, they can lie about their condition, fake and claim to be sick all because we can't see the patient. (TMP, 35 years)

#### Workload, staffing constraints and responsiveness challenges.

Providers further reported that responsiveness of services were affected by high client volumes and limited staffing across digital platforms. They particularly noted that peak usage periods often led to delayed response times especially on platforms with higher user engagement and limited personnel. They also further noted that these delays were a common source of user dissatisfaction. Quantitative findings corroborated this, with 32% of online FP users reporting long wait times when trying to connect with an e-pharmacist.

The waiting time is something most of them complained about. The customer may not get [a] quick response while using the Google chat of the e-pharmacy platform, oftentimes prefer [preferring] the WhatsApp platform for engagement. (36 years)

#### Inventory and logistics issues

Service providers reported that frequent inventory-related challenges, including product stock-outs and sometimes delays in delivery and disrupted service continuity. These logistical constraints were noted as a recurring issue in qualitative interviews. Quantitative data further supported these observations, with 28% of online FP users identifying product unavailability as a key challenge when attempting to access services through digital platforms.

The unavailability of drugs at our stores…before you get that information about the availability and price of the product, the customer has been waiting for, let's say 30 to 45 minutes, and they are already impatient compared to when a pharmacist is in front of you for in-stores pharmacy. (e-Pharmacist, 33 years)

#### Continuity of care and follow-up limitations

Providers highlighted difficulties in maintaining continuity of care, particularly when clients were referred to physical facilities for services that could not be completed online. Follow-up was often inconsistent, with many users failing to complete referrals. This loss to follow-up was attributed to factors such as additional transportation or service costs, the need for spousal consultation, or declining user engagement after the initial virtual interaction.

Sometimes the patient says…. they have to discuss with [their] husband… and then they go, and we don't hear from them again. (Telemedicine Provider, 28 years)

#### Policy and regulatory environment

The absence of clear and supportive regulations for digital health was identified as a limiting factor in the expansion and integration of digital platforms into mainstream FP services. Participants from both user and provider groups noted that regulatory uncertainty hindered innovation, investment, and collaboration with formal health systems

### Triangulation of findings (quantitative and qualitative)

To synthesize the patterns emerging across both quantitative and qualitative strands, Table 5 provides a triangulated summary of key motivators and barriers influencing uptake of digital FP platforms, highlighting areas of convergence and divergence across user perspectives.

**Table 5.** Integrated summary of quantitative and qualitative findings on use of digital health platform for family planning.

Theme	Quantitative evidence	Qualitative insight
Awareness of e-Pharmacy and Telemedicine	78% aware of e-pharmacy; 24% had used it. Telemedicine awareness lower (30% general, 6% FP-specific).	Participants knew about e-pharmacy but less about contraceptive availability; telemedicine rarely known for FP.
Use by Subgroup	Higher use among women, 18–26 years, singles, educated, higher-income groups (*p < *.001); Telemedicine linked to post-secondary education and higher income (*p < *.05).	Younger users valued privacy, ease of access, and stigma avoidance.
Products Accessed	Pills (63%), condoms (42%), emergency pills (36%); injectables (9%) and implants (17%) rarely accessed.	Long-acting methods perceived to require physical procedures.
Motivators for Use	72% convenience, 35% product access, 13% cost-saving.	Convenience, discretion, and online educational resources were key motivators.
Privacy and Stigma Avoidance	Singles more likely to use e-pharmacy (*p < *.001).	Discretion valued to avoid embarrassment of purchasing in-person.
Barriers	47% delivery delays, 33% distrust, 64% network issues, 28% product unavailability.	Users faced app navigation issues, trust concerns, courier delays, and preference for in-person care.
Trust and Credibility Concerns	33% expressed distrust in online services.	Concerns about counterfeit drugs and provider qualifications limited uptake.
Provider and System Challenges	32% reported long wait times connecting to providers.	Providers reported overload, poor rapport without nonverbal cues, drop-offs after referrals.
Continuity of Care	Not quantified.	Frequent user drop-offs when referred for physical consultations.
Mismatch Between Expectations and Scope	Telemedicine rarely used for FP initiation.	Users expected full-service FP online, unaware of need for in-person follow-up.

## Discussion

This mixed-methods study revealed a complex interplay of factors influencing the uptake of digital health platforms for FP in an urban Nigerian context. Our findings show that while enabling technology (smartphones, internet access) is increasingly present, mere availability of technology does not guarantee adoption of e-pharmacy or telemedicine for FP services. There exists a clear “digital divide” in who uses these innovations. Younger, more educated, and higher-income individuals are far more likely to be aware of and engage with digital health for FP. Older, less-educated, and lower-income individuals largely continue to rely on traditional in-person services. This is consistent with findings from other LMICs, where socioeconomic and demographic factors strongly shape digital health utilization patterns.^31,32^ In similar studies in East and West Africa, higher education and urban residency were consistently associated with greater use of mHealth interventions for reproductive health.^31,32^ Thus, despite Nigeria's growing internet penetration, digital FP services may currently be exacerbating inequities by primarily serving those already relatively advantaged. Bridging this gap will require targeted efforts to reach and support underrepresented groups, such as women in low-resource settings or with lower literacy, to also benefit from these platforms.

A prominent finding in our study is the low awareness and persistent misconceptions about digital FP options among the study participants, particularly regarding telemedicine. This is consistent with observations from other studies in Nigeria and in other LMIC contexts. Studies have noted that awareness of telemedicine in Africa remains low and often limited to those who had exposure during the COVID-19 pandemic or via donor-funded programs.^33,34^ In our study, many participants had little or no prior knowledge of telemedicine. Indeed, a pre-pandemic survey in Nigeria found that 59% of people had never heard of telemedicine.^35^ Moreover, participants’ concerns about using contraceptives without direct medical supervision reflect broader challenges in FP uptake reported in the literature. Myths about contraceptive side effects and long-term fertility impact are common in Nigeria and other parts of West Africa and have been shown to deter women from seeking FP information or services, whether online or offline. A multi-country study found that 50–70% of women believed that contraceptives could cause serious health risks or infertility.^36^ These patterns suggest that barriers to digital health adoption often transcend national boundaries, rooted in cultural norms, health literacy, and trust in healthcare systems.

In societies where face-to-face interactions are traditionally valued in healthcare, as in Nigeria, it is unsurprising that many participants voiced a preference for in-person consultations. This underscores the universal importance of trust and rapport in healthcare, regardless of delivery platform. People need to feel that their provider understands them, and for many, this trust is built more easily in person. The preference for in-person care may stem from deeply ingrained beliefs about the necessity of physical presence for effective healthcare and a fear that digital interactions might be depersonalized. Our data illustrate that older individuals and those less comfortable with technology often felt a sense of disconnect with online services, aligning with what Nicolini describes as the “stretching out” of work practices in telemedicine.^37^

Technical challenges also emerged as a critical barrier, consistent with prior research on mHealth in developing countries. Poor network connectivity, limited language support, and cumbersome user interfaces were all reported by users as hindrances to adoption.^38,39^ This finding aligns with systematic reviews highlighting unreliable infrastructure and low e-health literacy as frequent obstacles to mobile health uptake in low-resource environments.^38^ Even though participants possessed devices, unstable internet access and confusing app layouts reduced their willingness or ability to use services. Expanding technology access alone is insufficient; platforms must also be designed to be user-friendly and culturally relevant to diverse LMIC users.⁸ Improving network infrastructure will be critical to uptake. As long as dropping connections and slow speeds persist, user confidence in telehealth will remain fragile.

In addition to overcoming individual-level barriers, strengthening supply-side systems including regulatory frameworks, quality assurance mechanisms for digital platforms and provider capacity building initiatives are essential to sustain and scale digital FP services in urban Nigeria context and similar LMIC settings.

Privacy and trust issues identified in this study resonate with global concerns about digital healthcare. In many contexts, users fear that online transactions may not be secure or that personal health information could be misused. In our findings, trust issues particularly revolved around the authenticity of medications and the qualifications of online providers. Similar concerns were documented in Kenya, where confidentiality and service quality fears hindered digital FP information use among youth.^40^ Building trust will likely require regulatory oversight, visible endorsements by reputable health authorities, user education, and leveraging positive peer experiences. Our findings suggest that those who adopted e-pharmacy or telemedicine generally found value, implying that showcasing success stories could reassure hesitant users.

Applying the TAM, the perceived usefulness of e-pharmacy and telemedicine is evident to users. According to them it enabled convenience, privacy, and product options. However, perceived ease of use is hampered by technical glitches, and perceived uncertainty or risk remains high. Enhancing adoption thus requires strengthening perceived usefulness and ease of use while reducing uncertainty. This is consistent with findings from systematic reviews showing that trust significantly predicts acceptance of e-health technologies.^41^ Our recommendations on improving user interfaces, verifying provider credentials, and building trust align with these insights.

From the HBM perspective, our data suggest that perceived barriers (misinformation, network instability, safety concerns) often outweighed perceived benefits (convenience, privacy) for many potential users. Some women did not perceive themselves as needing digital channels if their current FP access was sufficient, consistent with low perceived susceptibility constructs within the HBM. Tailored education and system improvements could recalibrate perceptions and shift behavior.

The DOI theory offers further insights. E-pharmacy and telemedicine for FP in Lagos appear to be in the early adopter stage, primarily used by young, urban, tech-savvy populations. Broader adoption among the early majority will require reducing perceived risks, increasing trialability, and enhancing observability. Peer networks, community influencers, and user testimonials could serve as important catalysts. Current low telemedicine uptake indicates that it remains largely confined to innovators. The DOI suggests that public success stories, free trials, and visible benefits could help push these innovations into wider use.

More importantly, our findings align with broader LMIC literature indicating that many challenges to digital health are systemic. For instance, e-pharmacy regulatory gaps identified in this study are echoed across other low-resource settings, where technological advancement often outpaces regulatory systems.^42^ Quality control, prescription enforcement, and patient safety concerns require urgent attention. Similarly, sustaining the telemedicine expansion seen during COVID-19 requires greater integration into formal health systems and enhanced provider training.^43^ Our interviews with telemedicine providers reinforced this, highlighting the need for clear protocols, technical support, and capacity building.

Despite barriers, the potential benefits of digital FP platforms remain substantial. Even amidst challenges, motivated users valued the convenience, privacy, and flexibility offered. In a dense urban environment like Lagos, digital platforms could reduce overcrowding in clinics and offer discreet options for individuals facing stigma or time constraints. Early adopters can play a critical role in informing platform improvements and fostering wider adoption through peer influence.

Beyond Nigeria, evidence from this study have relevance for other LMICs grappling with similar reproductive health challenges. Scaling digital health for FP requires a holistic approach, combining demand generation (education, trust-building) with supply-side improvements (infrastructure, platform quality, regulatory oversight). Barriers such as limited awareness, technical issues, trust deficits, and privacy concerns are likely to be common across contexts, so interventions piloted in Nigeria could offer adaptable models elsewhere.

Aligning digital health efforts with Nigeria's FP2030 commitments and broader health sector digital transformation goals is crucial. Digital services should complement, not replace, traditional FP services. A hybrid model starting contraceptive journeys in clinics but facilitating follow-up through digital means could offer an optimal solution, as piloted in other FP programs globally.

Finally, while our data were not structured along a formal multilevel analytical framework, the findings reveal that key motivators and barriers to digital FP platform use operate across individual, household, and system levels. This underscores the need for integrated strategies that address personal digital readiness, household dynamics, and broader service delivery and regulatory environments to enhance equitable uptake and sustained use.

## Conclusions

This mixed-methods study provides valuable insights into the motivators and barriers to uptake and utilization of digital health platforms, specifically e-pharmacy and telemedicine, for FP in Lagos, Nigeria. We found that these innovative channels have significant potential to expand access to FP services, offering convenience and privacy that are highly valued by certain segments of the population (notably younger, educated, higher-income individuals). However, our findings also highlight that several barriers must be understood and addressed for the successful integration of e-pharmacy and telemedicine into the wider FP landscape.

### Study limitations

The urban, relatively literate sample limits generalizability to rural populations with lower digital access. The cross-sectional design of the study precludes definitive causal conclusions. Social desirability bias may have influenced self-reported awareness and usage. Telemedicine uptake was low, limiting regression analysis power for that subgroup. Nevertheless, the study's mixed-methods triangulation strengthens confidence in the findings, providing nuanced explanations for observed patterns. Another key limitation of this study is that the questionnaire used was not based on a previously validated tool. However, it was reviewed and refined with input from subject matter experts in FP and digital health to enhance its content validity.

### Practical implications and policy recommendations

Building on the study findings, several actionable strategies are proposed to strengthen the uptake of digital health platforms for FP services.

Demand-side Recommendations:

- Enhance public awareness: Implement targeted communication campaigns to increase knowledge about the availability, safety, and benefits of digital FP platforms.
- Improve digital literacy: Develop and support initiatives aimed at enhancing users’ ability to navigate digital health platforms, particularly among women with lower educational attainment and adolescents.
- Address affordability concerns: Explore subsidies, insurance partnerships, and cost-reduction strategies to make digital FP services more accessible to low-income users.
- Strengthen privacy assurances: Communicate clear, transparent data protection measures to users, emphasizing confidentiality and the security of personal health information.
- Tackle stigma and discrimination: Design culturally sensitive messaging and discreet access points to reduce social stigma, especially among unmarried women and adolescents.

Supply-side Recommendations:

- Expand service availability: Ensure a wide range of contraceptive products and services are available through digital platforms to meet diverse user needs.
- Strengthen platform infrastructure: Improve technological systems to minimize network disruptions, reduce service delivery delays, and enhance user experience.
- Train digital service providers: Invest in the training of online FP providers on client confidentiality, professional standards, and user-centered care.
- Enhance trust and credibility: Establish certification systems and visible quality assurance indicators for digital FP platforms to boost user confidence.
- Ensure continuity of care: Develop seamless referral systems that link digital services with physical healthcare providers to maintain care consistency when needed.

## Supplemental Material

sj-docx-1-dhj-10.1177_20552076251349624 - Supplemental material for Motivators and barriers to the uptake of digital health platforms for family planning services in Lagos, Nigeria: A mixed-methods study Supplemental material, sj-docx-1-dhj-10.1177_20552076251349624 for Motivators and barriers to the uptake of digital health platforms for family planning services in Lagos, Nigeria: A mixed-methods study by Mohammed M. Alhaji, Muhammad R. Balarabe, David Atama, Arizechukwu Okafor, Doluwamu Solana, Francis Meyo, Chinedu Joseph Okoye, Uchenna Okafor, Babatunde Oyelana and Jennifer Anyanti in DIGITAL HEALTH

sj-docx-2-dhj-10.1177_20552076251349624 - Supplemental material for Motivators and barriers to the uptake of digital health platforms for family planning services in Lagos, Nigeria: A mixed-methods study Supplemental material, sj-docx-2-dhj-10.1177_20552076251349624 for Motivators and barriers to the uptake of digital health platforms for family planning services in Lagos, Nigeria: A mixed-methods study by Mohammed M. Alhaji, Muhammad R. Balarabe, David Atama, Arizechukwu Okafor, Doluwamu Solana, Francis Meyo, Chinedu Joseph Okoye, Uchenna Okafor, Babatunde Oyelana and Jennifer Anyanti in DIGITAL HEALTH

sj-docx-3-dhj-10.1177_20552076251349624 - Supplemental material for Motivators and barriers to the uptake of digital health platforms for family planning services in Lagos, Nigeria: A mixed-methods study Supplemental material, sj-docx-3-dhj-10.1177_20552076251349624 for Motivators and barriers to the uptake of digital health platforms for family planning services in Lagos, Nigeria: A mixed-methods study by Mohammed M. Alhaji, Muhammad R. Balarabe, David Atama, Arizechukwu Okafor, Doluwamu Solana, Francis Meyo, Chinedu Joseph Okoye, Uchenna Okafor, Babatunde Oyelana and Jennifer Anyanti in DIGITAL HEALTH
