# Supplementary material for: Motivators and barriers to the uptake of digital health platforms for family planning services in Lagos, Nigeria: A mixed-methods study
Source: Digit Health. 2025 Jun 9;11:20552076251349624. doi: 10.1177/20552076251349624 (PMC12159478; doi:10.1177/20552076251349624)
Supplement: sj-docx-1-dhj-10.1177_20552076251349624 - Supplemental material for Motivators and barriers to the uptake of digital health platforms for family planning services in Lagos, Nigeria: A mixed-methods study [file sj-docx-1-dhj-10.1177_20552076251349624.docx]

# **COREQ Flowchart for the Qualitative Phase of the study**

| **Participants identified through e-pharmacies, telemedicine platforms, clinics, and referrals** (n = 92) |
| --- |
| ↓ |
| Excluded (n = 13) **Did not meet eligibility** (e.g., insufficient experience, not using digital FP platforms, declined consent after screening) |
| ↓ |
| **Eligible participants invited to participate (n = 79)** |
| ↓ |
| **Participants who consented and completed interviews (n = 79)** - End-users (n = 49) - Providers (n = 30) |
| ↓ |
| **Interviews conducted (n = 79)** - Conducted face-to-face or via secure video call - Conducted in English - Duration: 30–60 minutes - Audio-recorded and transcribed verbatim |
| ↓ |
| **Data analyzed (n = 79)** - Thematic analysis using NVivo - Themes and sub-themes developed inductively and deductively - Inter-coder reliability checks performed |
| ↓ |
| **Key themes developed and reported** - Motivators for use of digital platforms  - Demand-side Barriers to Uptake - Supply Side Barrier - Trust and Credibility Concern |

**Themes and Sub-Themes Developed from Thematic Analysis**

1. **Motivators for Use of Digital Platforms**
   - Convenience and ease of access
   - Privacy and confidentiality
   - Cost savings and affordability
   - Access to a wider range of family planning products and services
2. **Demand-side Barriers to Uptake**
   - Limited awareness of digital FP platforms
   - Digital literacy challenges
   - Financial affordability barriers
   - Privacy and confidentiality concerns
   - Stigma and fear of social judgment
3. **Supply-side Barriers to Uptake**
   - Network and internet connectivity challenges
   - Service delivery delays and provider overload
   - Product stock-outs and availability limitations
   - Challenges with prescription verification
4. **Trust and Credibility Concerns**
   - Fears about counterfeit or substandard products
   - Doubts about provider authenticity and professionalism
   - Concerns about the confidentiality of consultations
