# Supplementary material for: Motivators and barriers to the uptake of digital health platforms for family planning services in Lagos, Nigeria: A mixed-methods study
Source: Digit Health. 2025 Jun 9;11:20552076251349624. doi: 10.1177/20552076251349624 (PMC12159478; doi:10.1177/20552076251349624)
Supplement: sj-docx-2-dhj-10.1177_20552076251349624 - Supplemental material for Motivators and barriers to the uptake of digital health platforms for family planning services in Lagos, Nigeria: A mixed-methods study [file sj-docx-2-dhj-10.1177_20552076251349624.docx]

**Qualitative In-Depth Interview Guide**

**Protocol and Instructions to Interviewer**

Method: In-depth interviews

Tools: Interview guides, audio recorder, field notes

Target participants: Current users of an E-Pharmacy platform (Family Planning and non-Family Planning)

Time: About 60 minutes

Language: English

**Instructions**

1. Clearly label your notes and your recordings to be able to find them easily during the analysis.
2. Cover all topics and use your judgment throughout to answer the objectives for each section. The questions and structure below are a guide to help answer these objectives but can be deviated from if you believe that the objectives can be better met another way. Do not feel like you must read out the suggested questions.
3. Use probes and follow-up questions to steer the interview with the respondent’s own narrative.
4. Jot notes as you go and don’t forget to write down memorable quotes.
5. Circle notes that discuss similar themes.
6. When writing notes, in addition to the participant's response, note down anything surprising about the interviewee’s reaction to some questions or tone of response (e.g. laughing, getting uncomfortable, distressed etc.).
7. Always probe for more. Get examples and details. First answers are often generalities.
8. Repeat statements to confirm you’ve understood correctly.
9. Build rapport -by not expressing judgment and communicating to participants that they are the experts of their own lives. Think about how you can help them feel comfortable answering sensitive questions.
10. Never answer a question for the respondent.
11. Try not to think about time - relax into the interview and give the participants enough time to respond to your questions.
12. Make sure to mention in the introduction that it is okay for the participants to stop the interview at any time. If they do need to stop the interview, ask them if they are willing to participate on another day. Do the same if the participant seems **rushed or short** on time.

**Introduction**

# **Purpose: Why are we doing this research?**

Thank you for taking the time to speak with us today. My name is <*insert FO name*> from Busara Center for Behavioral Economics, a research company based in Kenya. I would like to invite you to participate in this study

This study is looking to understand the needs, decision-making processes, barriers, and facilitators for the users of the HealthPlus e-Pharmacy and telehealth platform to access Family Planning counseling, products, services in Lagos, Nigeria. We are interested in hearing from you as a current user of the platform and we believe that your participation in this study will generate important insights that will be invaluable to our study.

An important reminder:

Your participation is entirely voluntary, meaning you may withdraw from this study at any time without penalty. If you withdraw mid-interview, we will destroy all the interview data collected up till that point. If you wish to withdraw and want all your data destroyed, you may contact the research team and request this from any device. If you volunteer to participate in this study, we will ask you to provide some personal information including your first name, your phone number, and some demographic details. Any information you provide will be kept confidential, meaning no one else will be able to access it.

The interview will last approximately 30 to 60 minutes. We would also like to record the interviews so we can refer to what you said when we are writing our report.

We would now like to start recording this interview, including as we go through each line of consent for the study. Are you okay with us recording the rest of this interview?

**Qualitative In-Depth Interview Guide – E-Pharmacy Current Users**

**Introduction**

| **Background Information** |
| --- |
| Name of the interviewer: _________________________________________________________  Date of the interview (dd/mm/yy): _________________________________________________  Location of the interview: ________________________________________________________ |

| **Respondent Demographics** |
| --- |
| - Name of the interviewee: _________________________________________________________ - Assigned PID: ___________________________________________________________________ - Age (in years): ___________________________________________________________________ - Occupation: _____________________________________________________________________ - LGA/State: ___________________________________________________________________ - Highest level of education (please select one):   - No education   - Completed Primary School   - Completed Secondary School   - Completed Islamic School   - Diploma/Certificate   - Undergraduate Degree   - Postgraduate Degree   - Other, please specify: ______________________________________________________ - Device ownership (select multiple if necessary):   - I own a smartphone/tablet   - I own a computer   - I have access to a smartphone/tablet but do not own it   - I have access to a computer but do not own it   - I do not have access to either a smartphone, tablet or computer - Have you used an e-Pharmacy Platform?   - Yes - for products and services **including** Family Planning Services   - Yes - for products and services **except** Family Planning Services   - No - Relationship Status:   - Single   - Married   - Divorced   - Living together (but not married)   - Other ( specify):___________________ - Religion:   - Christianity   - Islam   - Traditional   - Others (specify)___________________ - Average monthly iIncome level (select one):   - Under N 50,000   - N 50,000 to N 150,000   - Above N 150,000 |

| **Awareness and uptake of online pharmacy services** |
| --- |
| 1. What online/e-pharmacies in Lagos do you know of where you can buy FP medications? Please list them.    1. Have you ever used any of these online pharmacy/e-pharmacy platforms? Why/Why not? 2. How did you learn about this e-pharmacy platform?    1. Do you know others who use e-pharmacy?   Probe: What types of people use e-pharmacy?   1. What made you start using the e-pharmacy platform?    1. How frequently do you use the platform?    2. What gadgets do you use to access the e-pharmacy platform (probe laptop or smartphone and ownership)       1. If **smartphone**: ask to whether it is through android or apple Apps, or mobile web    3. What types of products do you often buy online and why do you buy them online instead of in stores?    4. How do you get the prescriptions for some of the prescription-only medications?    5. Do you have your products delivered to your home or to a pick up point? Why?    6. What is the average delivery time from when you paid to when it is delivered to you?       1. Are you satisfied with the delivery process and time?       2. If not, what do you suggest to be done to improve the experience? 2. On a scale of 1 to 5 (where 1 is Very easy, 2 Easy, 3 Neutral, 4 Difficult and 5 Very difficult) how easy/difficult has it been using the e-pharmacy platform?    1. Why did you give this answer? 3. What do you like most about the e-pharmacy website and services?   Probe about:   - Product cost, - Product variety/availability - Product quality - Delivery cost/speed/availability - Website visuals and ease of website navigation - Online shopping support - Ease of checkout/payment, other.   1. Why did you give this answer?  1. What do you dislike about the e-pharmacy website and services?   Probe about:   - Product cost, - Product variety/availability - Product quality - Delivery cost/speed/availability - Website visuals and ease of website navigation - Online shopping support - Ease of checkout/payment, other   1. Why did you give this answer?  1. Briefly describe your experience purchasing or accessing product/service on the e-platform.   Probe about experience when:   - Uploading subscriptions/prescriptions - Engaging with a pharmacist via the live chat - Engaging with the call center - Connecting with Mutti Doctor for remote/virtual physical examination or prescription  1. In your opinion, what can be done to improve the e-pharmacy experience?    1. Are there any other services/features you would like to have on the platform? What are they?    2. What would make you do all of your pharmacy shopping through an e-pharmacy? 2. How do you pay for products online (for example, bank deposits/transfers, banking apps, credit/debit cards, online payment portals such as PayStack, ATM transfer, through POS terminal agents, PayPal, mobile money services, other payment methods)?    1. In your community, what are other common payment methods for online purchases (refer to list above)?    2. In your opinion, what are the challenges with different payment mechanisms (for example, what might prevent others from accessing these mechanisms)? 3. Besides the e-pharmacy you currently use, what online pharmacy platforms or e-commerce platforms do you know about (Konga, Jumia, MedPlus, etc.)?    1. Which of these have you used before and why?    2. What types of products did you buy? 4. In your opinion, how is the e-Pharmacy platform you use better or worse when compared to other online shopping platforms?   Probe about:   - Product cost - Product variety/availability - Product quality - Delivery cost/speed/availability - Website visuals and ease of website navigation - Online shopping support - Ease of checkout/payment, other  1. What do you think are the top three barriers to using e-pharmacy for other people in Lagos?   Probe:   - Lack of awareness of e-pharmacy - Lack of capacity/literacy to use online services - Cost of products - Cost of delivery/shipping - No access to payment mechanisms - Privacy concerns - Access to internet/smartphone/computer - Availability of delivery coverage, i.e., areas not supported by delivery services, other.  1. What questions do you have about purchasing Family Planning products from an e-pharmacy?   **Note to FO**: Collect as many questions as possible but let the participant know you are only collecting questions and are not in a position to provide answers to them now. These questions will be used to provide answers to the general public in the future |

| **Awareness and uptake of telemedicine** |
| --- |
| 1. In your own words, can you briefly tell me what telemedicine is?   FO Instructions: If the respondent appropriately defines “telemedicine”, move on to the next question.  FO Instructions: If the respondent **does not** appropriately define “telemedicine”, please read the following:  *“Telemedicine is the provision of healthcare services and information virtually, through digital platforms. An example of this is having a video appointment/ consultation a with doctor, nurse, or pharmacist where they can assess your health and well-being and provide advice, prescriptions, and/or referrals for in-person care.,*   1. What telemedicine providers have you heard of in Lagos, if any? Please list them out.    1. How did you learn about this/these telemedicine provider(s)? 2. Have you ever accessed telemedicine services?    1. If yes, what types of telemedicine services did you receive and why?       1. What device did/do you use to access telemedicine services?   Probe for device use: Phone or computer   - Owned or shared devices   - 1. How frequently do you use this/these service(s)?     2. What was your experience using this/these service(s)?   Probe:   - What did they like or dislike about using the telemedicine platform? - Was the platform easy to use? - Did they receive the care they needed through the platform?   1. If no, why not?      1. What might make telemedicine services interesting to you in the future?   2. Do you know anyone (family/friends) who use telemedicine services?      1. If yes, do you know what services they use and what were their experiences?  1. What is your opinion on the benefits and challenges of receiving healthcare/medical counsel virtually via your phone/computer?    1. Would you be interested in getting prescriptions virtually? – Why or why not? 2. What else would you consider when accessing telemedicine services?   Probe:   - Privacy - Confidentiality - Data security - Cost - Convenience  1. Are there any commonly-held beliefs about accessing online pharmacy services or telemedicine in your community?    1. If yes, what are they? 2. Would you feel comfortable talking to a qualified telemedicine provider about Family Planning (for example, what contraceptive methods might best support your pregnancy intentions)?    1. Why or why not? 3. What questions do you have about getting Family Planning counseling, prescriptions, and services from a telemedicine provider?   **Note to FO**: Collect as many questions as possible but let the participant know you are only collecting questions and are not in a position to provide answers to them now. These questions will be used to provide answers to the general public in the future |

| **Awareness and uptake of Family Planning Product and Services** |
| --- |
| 1. Can you please describe, in your own words, what "Family Planning" is? 2. What Family Planning products do you know about? Can you list them?    1. Which products do you use currently/have used in the past? Why?    2. Where do you currently access or purchase the Family Planning products from?    3. Why is this your preferred method of purchasing Family Planning products?       1. If via online/e-pharmacies, what do you consider when deciding to access Family Planning products via online platforms? 3. Do you decide alone or with your significant partner or someone else on whether and where you purchase Family Planning products and services or do others make the decisions?    1. If “**others**”: Who, and do you think that they would be interested in learning more about accessing Family Planning products and services online?       1. Why or why not? 4. What other support, if any, do you need to use a Family Planning method of your choice? 5. Where do you, your friends, and others in your community usually access Family Planning products and services?   Probe for sources:   - Public health facility - Private health facility - Pharmacy - Somewhere else.   1. Why?  1. Where do you, your friends, and others in your community usually access Family Planning information and counseling?   Probe for:   - Health facilities - Pharmacies - Internet/online services - Social media/apps - Traditional media: Radio/TV - Somewhere else   1. Are these sources trustworthy? Why/why not?   2. Are you satisfied with the information they provide? Why/why not?  1. Have you ever looked for information about Family Planning online?    1. What types of questions did you have?    2. What sources did you use (general Google search, specific website or app, other online source)?    3. On a scale of 1 to 5 (where 1 is “*Not at all trustworthy*” and 5 is “*Extremely trustworthy*”), how much do you trust this/these source(s)?    4. How helpful was it?    5. What issues, if any, did you experience accessing information online? 2. Have you ever received any Family Planning counseling from an e-pharmacy or telehealth provider?    1. If not, would you be interested in receiving FP counseling from an e-pharmacy or telehealth provider – Why or why not? 3. How do you think accessing Family Planning products via the e-Pharmacy platform would compare with accessing them at the brick-and-mortar stores?    1. Which of these options do you prefer to access FP products and why?    2. Does it depend on the FP method? What are the different considerations for getting oral pills vs. injectable, vs. an implant online?    3. What are the key factors that influence this choice?   Probe about:   - - 1. Structural barriers (access to internet, ownership of a smartphone, location of physical pharmacy, distance from home, privacy/confidentiality, data limitations, network issues/technical issues, delivery costs)     2. Psychological barriers (effort of learning new technologies, attitude of pharmacy staff members, concerns of online privacy)  1. If you could order an implant or an injectable online and set up an appointment for administration in the pharmacy, would this be something you would consider doing? Why or why not? 2. Have you ever used the Sayana Press/DMPA-SC? 3. What are the commonly-held beliefs in your community about Family Planning that may affect uptake of Family Planning services?    - 1. How do these beliefs affect your use of Family Planning?      2. Do you/your family members hold any of these beliefs? |

| **DMPA-SC/ Sayana Press - Only for those who have previously used Sayana Press** |
| --- |
| 1. Can you please tell me about your previous use of Sayana Press (DMPA-SC)?    1. Why did you choose this method for yourself?    2. What do you like about this method?    3. What do you dislike about this method?    4. Can you please tell me about your experience when you received it?   Probe:   - What type of facility/provider was it? - Did the provider tell you that you had the option to self-inject and train you on how to do it?  1. **FO Instructions**: If the respondent has **had** SELF INJECT TRAINING ask the following, otherwise move to the next question:    1. What do you think about the ability to self-inject?    2. What do you like/dislike?    3. Can you please tell us about the training you received for self-injection?    4. Did the training make you feel confident that you could self-inject? Why or why not?    5. What did the provider do to make you feel confident that you could self-inject? 2. **FO Instructions**: If the respondent has **not had** SELF-INJECT TRAINING was provided, read the following:   *“There is a new option for users of Sayana Press to learn how to self-inject from a provider, and once you have proven that you can safely self-inject, you are able to take refills home with you and you do not have to return to the provider for your next injection.”*   - 1. Do you think that the self-inject option would be interesting for you or others? Why or why not?   Probe:   - What might be the benefits of self-injection? - What might be the challenges?  1. If self-injection training was available through telemedicine, for example through a live video call with a trained provider where they would show you how to do it, and watch you practice, and then do it, how do you think receiving these instructions virtually would compare to receiving training from a provider in-person?    1. Why? 2. What concerns do you have about self-injections through a telemedicine or an e-pharmacy platform?   Probe for:   - Concerns about the accuracy of the information you receive remotely - Concerns about safety/effectiveness of self-administering DMPA-SC self-inject, etc  1. What additional support or information would you need in order to feel comfortable with self-administering DMPA-SC through a telemedicine or an e-pharmacy platform?   Probe about:   - Demonstrations or instructions of how to administer the medication - Access to healthcare provider to answer any questions or concerns, etc  1. If you have already received your self-injection training in-person, would you be interested in **telemedicine** support for your next self-injection?    1. Please explain your answer    2. Would you prefer to do your next self-injection alone or in-person with a healthcare provider?    3. Please explain why you have this preference    4. What benefits or challenges do you see with getting telemedicine support for your subsequent self-injections?    5. What kind of information would you like to access about the Sayana Press via telemedicine/e-pharmacy platforms?    6. How would you like this information to be delivered to you?   Probe for preferences for teleconsultations, online banners, infographics, email messaging, online testimonials or something else.   - 1. Why did you give this answer?  1. Are there cultural or social beliefs that might affect your willingness or ability to self-inject with the support of telemedicine?    1. Please explain your answer, if yes. 2. Do you have concerns about how your family or community might react to your self administering the medication? 3. What questions do you have about getting training or support from a telemedicine provider for self-injection?   **Note to FO**: Collect as many questions as possible but let the participant know you are only collecting questions and are not in a position to provide answers to them now. These questions will be used to provide answers to the general public in the future. |

**Qualitative Guide – Brick-and-Mortar / Potential e-pharmacy Users**

**Introduction**

| **Background Information** |
| --- |
| Name of the interviewer: _________________________________________________________  Date of the interview (dd/mm/yy): _________________________________________________  Location of the interview: ________________________________________________________ |

| **Respondent Demographics** |
| --- |
| - Name of the interviewee: _________________________________________________________ - Assigned PID: ___________________________________________________________________ - Gender (please select one):   - Female   - Male   - Prefer not to say - Age (in years): ___________________________________________________________________ - Occupation: _____________________________________________________________________ - Country: ________________________________________________________________________ - LGA/State: ___________________________________________________________________ - Highest level of education (please select one):   - No education   - Completed Primary School   - Completed Secondary School   - Completed Islamic School   - Diploma/Certificate   - Undergraduate Degree   - Postgraduate Degree   - Other, please specify: ______________________________________________________ - Device ownership (select multiple if necessary):   - I own a smartphone/tablet   - I own a computer   - I have access to a smartphone/tablet but do not own it   - I have access to a computer but do not own it   - I do not have access to either a smartphone, tablet or computer - Have you used the HealthPlus e-Pharmacy Platform?   - Yes - for products and services **including** Family Planning Services   - Yes - for products and services **except** Family Planning Services   - No - Relationship Status:   - Single   - In a relationship   - Married   - Cohabiting (living but unmarried) - Religion:   - Christianity   - Islam   - Traditional   - Others (specify)_______________ - Number of dependent children: ____________________________________________________ - Income: (select one):   - Under N 50,000   - N 50,000 to N 150,000   - Above N 150,000 |

| **Knowledge of telemedicine providers and services** |
| --- |
| 1. In your own words, can you briefly tell me what telemedicine is?   **FO Instructions**: If the respondent appropriately defines “telemedicine”, move on to the next question.  **FO Instructions**: If the respondent does **not** appropriately define “telemedicine”, please read the following out loud and ask the question below:  "*Telemedicine is the provision of healthcare services and information virtually through digital platforms. An example of this is having a video appointment.consultation with a doctor, nurse or pharmacist where they can assess your health and well-being and provide advice, prescriptions, and/or referrals or in-person care.*"   1. What telemedicine providers have you heard of in Lagos, if any? Please list them out.    1. How did you hear about this/these telemedicine provider(s)? 2. Have you accessed telemedicine services before?    1. If yes, what types of telemedicine services have you received?       1. What device did/do you use to access telemedicine services? And why?   Probe for device use: Phone or computer   - Owned or shared devices   - 1. How frequently did/do you use these service(s)?     2. What was your experience using this/these service(s)?   Probe for:   - What did they like/dislike about using the telemedicine platform? - Was the platform difficult/easy to use? - Did they receive the care they needed through the platform?   1. If no, why not?      1. What might make telemedicine services interesting to you in the future?   2. In your opinion, how do you think accessing telemedicine would compare to accessing healthcare in person at a facility?      1. Which form of healthcare delivery are you more comfortable with? Why?  1. What is your opinion on the benefits and challenges of receiving healthcare/medical counsel virtually via your phone/computer?    1. Would you be interested in getting prescriptions virtually?    2. Why/why not? 2. Would you feel comfortable talking to a qualified telemedicine provider about Family Planning (for example, what contraceptive methods might best support your pregnancy intentions)?    1. Why/why not? 3. How would your friends, family or others in your community see getting Family Planning counseling and contraceptive prescriptions, if needed, from a qualified telemedicine provider?    1. Why/why not? 4. What else would you consider when accessing telemedicine services?   Probe about:   - Privacy/confidentiality - Data security - Cost - Convenience  1. What are commonly-held beliefs about accessing telemedicine in your community?    1. What is your view of these beliefs? 2. What questions do you have about Family Planning, prescriptions and services from a telemedicine provider?   **Note to FO**: Collect as many questions as possible but let the participant know you are only collecting questions and are not in a position to provide answers to them now. These questions will be used to provide answers to the general public in the future. |

| **Awareness and uptake of e-pharmacy services** |
| --- |
| 1. Can you briefly tell me what you know about e-pharmacy or online pharmacy?   **FO Instructions:** If the respondent appropriately describes “e-pharmacy / online pharmacy”, move on to the next question  **FO Instructions**: If the respondent **does not** appropriately describes “e-pharmacy platform”, read the following out loud:  "An e-pharmacy is a service that allows people to purchase their prescribed or over-the-counter medication and health products online to be delivered to their homes.”   1. Note to FO: Please read this scenario to the participants then proceed to ask questions. “Now I want to introduce you to Halima, a 34 years old woman residing in Lagos. She is interested in using e-pharmacy to purchase her family planning medications.” 2. What online/e-pharmacies in Lagos do you know of where Halima can buy FP medications? Please list them.    1. Have you ever used any of these online pharmacy/e-pharmacy platforms? Why/Why not?    2. What challenges will Halima likely experience when using an e-pharmacy platform to purchase the FP medication?   Probe:   - Lack of awareness of e-pharmacy - Lack of capacity/literacy to use online services - Cost of products - Cost of delivery/shipping - No access to payment mechanisms - Privacy concerns - Access to internet/smartphone/computer - Availability of delivery coverage, i.e., areas not supported by delivery services, etc   1. How can we encourage women like Halima to overcome the challenges identified above?   2. Have you personally experienced any of these challenges before? How did you deal with them?  1. What can be done to encourage you to use an e-pharmacy platform?    1. Would you consider purchasing Family Planning products through an e-pharmacy in the future? Why/why not? 2. Do you typically order for anything online (e.g., food) and have it delivered to you at home?   **Note to FO**: If participant says “yes”, probe for:   - The specific items - and how recent was the purchase and whether this is routine for them? - How much did they paid for the delivery (other the cost of the items) - Specific concerns or challenges they have experienced in online orders  1. How would you like to pay for home or nearby deliveries and pickups of your medications?   Probe for: cash, USSD transfer, POS terminal or other payment methods   - 1. What would make you consider the option of getting your medications delivered to you at home?   Probe for:   - Specific health services/product they are willing to pay for home delivery - Which would they prefer - pay before delivery or pay on delivery? - Maximum amount they are willing to pay for delivery services  1. What questions do you have about purchasing Family Planning products from an e-pharmacy?   **Note to FO**: Collect as many questions as possible but let the participant know you are only collecting questions and are not in a position to provide answers to them now. These questions will be used to provide answers to the general public in the future.   1. In your opinion, how does accessing Family Planning products via an e-pharmacy/online pharmacy platform compare with accessing them at a physical pharmacy?    1. Which of these two do you prefer to access pharmacy services?    2. What might encourage you to use an on-line pharmacy instead of a physical pharmacy? |
| **Perception on the HealthPlus e-Pharmacy Platform for FP products** |
| **Note to FO**: Read aloud: “***The HealthPlus e-pharmacy platform is a digital platform that allows customers and users access health products such as over-the-counter or prescription medicines and health products, and services such as consultations with health practitioners.”***   1. What do you think of the HealthPlus e-pharmacy platform?    1. In what way do you think accessing FP products and services via the platform may be different from accessing these services at the physical brick-and-mortar pharmacies?    2. How might people living in your community be engaged to encourage them to use the HealthPlus e-pharmacy platform? 2. What might keep you from using the Healthplus e-pharmacy platform? Please state as many as you can think of.    1. Would you consider purchasing your FP products on this platform? Why or why not?    2. If you were to decide to access FP services via the platform, what information would you need to use this platform? Why is this important?    3. What can be done, in your opinion, to encourage more people in your community to use the Healthplus e-pharmacy platform to access FP information, products and services? 3. Are there commonly-held beliefs about e-pharmacy services in your community? If yes, what are they? 4. What questions do you have about purchasing Family Planning products from an e-pharmacy?   **Note to FO**: Collect as many questions as possible but let the participant know you are only collecting questions and are not in a position to provide answers to them now. These questions will be used to provide answers to the general public in the future |

| **Awareness and uptake of Family Planning** |
| --- |
| 1. To start with, can you please describe, in your own words, what "Family Planning" is?    1. Is Family Planning something that is relevant to your life currently? Why/why not?    2. What are the commonly-held beliefs about Family Planning that may affect the uptake of Family Planning services in your community?       1. Do you/your family members hold any of these beliefs?       2. How do these beliefs affect your use of Family Planning? 2. Suppose you or your friends in Lagos are interested in family planning products and services. Where would they access Family Planning products and services?   Probe for sources:   - Public health facility - Private health facility - Pharmacy - Somewhere else   1. Why would these be the preferred options?  1. Now, suppose your or your friends in Lagos are interested in information on FP and counseling? Where would they access this information??   Probe for:   - Health facilities - Pharmacies - Internet/online services - Social media/apps - Traditional media: Radio/TV - Somewhere else   1. Are these sources trustworthy? Why/why not?   2. Are you satisfied with the information they provide? Why/why not?  1. Have you ever looked for information about Family Planning online?    1. What types of questions did you have?    2. What sources did you use (general Google search, specific websites or apps, any other online source)?    3. On a scale of 1 to 5 (where 1 is “*Not at all trustworthy*” and 5 is “*Extremely trustworthy*”), how much do you trust this/these source(s)?    4. How helpful was it?    5. What issues, if any, did you experience accessing information online? 2. Would you be interested in receiving Family Planning counseling from an e-pharmacy or telemedicine provider?    1. Why/why not? 3. What Family Planning products do you know about? Can you list them?    1. Which products do you use currently/have used in the past? Why?    2. Where do you access Family Planning products?    3. Why is this your preferred method of accessing Family Planning products? 4. What do you consider when deciding to access Family Planning products via online platforms?    1. How do these factors affect your ability to access Family Planning products? 5. Are you the only person who decides whether and where you purchase Family Planning products and services or do others make the decisions?    1. If “others”: who are these people?       1. Do you think they would be interested in learning more about accessing Family Planning products and services online?       2. Why/Why not? 6. What other support, if any, do you need to use a Family Planning method/product of your choice? 7. Have you ever used the Sayana Press/DMPA-SC? |

| **DMPA-SC/ Sayana Press - Only for those who have previously used Sayana Press** |
| --- |
| 1. Can you please tell me about your previous use of Sayana Press (DMPA-SC)?    1. Why did you choose this method for yourself?    2. What do you like about this method?    3. What do you dislike about this method?    4. Can you please tell me about your experience when you received it?   Probe:   - What type of facility/provider was it? - Did the provider tell you that you had the option to self-inject and train you on how to do it?  1. **FO Instructions**: If the respondent has **had** SELF INJECT TRAINING ask the following, otherwise move to the next question:    1. What do you think about the ability to self-inject?    2. What do you like/dislike?    3. Can you please tell us about the training you received for self-injection?    4. Did the training make you feel confident that you could self-inject? Why or why not?    5. What did the provider do to make you feel confident that you could self-inject? 2. **FO Instructions**: If the respondent has **not had** SELF-INJECT TRAINING was provided, read the following:   *“There is a new option for users of Sayana Press to learn how to self-inject from a provider, and once you have proven that you can safely self-inject, you are able to take refills home with you and you do not have to return to the provider for your next injection.”*   - 1. Do you think that the self-inject option would be interesting for you or others? Why or why not?   Probe:   - What might be the benefits of self-injection? - What might be the challenges?  1. If self-injection training was available through telemedicine, for example through a live video call with a trained provider where they would show you how to do it, and watch you practice, and then do it, how do you think receiving these instructions virtually would compare to receiving training from a provider in-person?    1. Why? 2. What concerns do you have about self-injections through an e-pharmacy platform?   Probe for:   - Concerns about the accuracy of the information you receive remotely - Concerns about safety/effectiveness of self-administering DMPA-SC self-inject, etc  1. What additional support or information would you need in order to feel comfortable with self-administering DMPA-SC through a telemedicine or an e-pharmacy platform?   Probe about:   - Demonstrations or instructions of how to administer the medication - Access to healthcare provider to answer any questions or concerns, etc  1. If you have already received your self-injection training in-person, would you be interested in telemedicine support for your next self-injection?    1. Please explain your answer    2. Would you prefer to do your next self-injection alone or in-person with a healthcare provider?    3. Please explain why you have this preference    4. What benefits or challenges do you see with getting telemedicine support for your subsequent self-injections?    5. What kind of information would you like to access about the Sayana Press via telemedicine/e-pharmacy platforms?    6. How would you like this information to be delivered to you?   Probe for preferences for teleconsultations, online banners, infographics, email messaging, online testimonials or something else.   - 1. Why did you give this answer?  1. Are there cultural or social beliefs that might affect your willingness or ability to self-inject with the support of telemedicine?    1. Please explain your answer, if yes. 2. Do you have concerns about how your family or community might react to your self administering the medication? 3. What questions do you have about getting training or support from a telemedicine provider for self-injection?   **Note to FO**: Collect as many questions as possible but let the participant know you are only collecting questions and are not in a position to provide answers to them now. These questions will be used to provide answers to the general public in the future. |
