# Supplementary material for: Motivators and barriers to the uptake of digital health platforms for family planning services in Lagos, Nigeria: A mixed-methods study
Source: Digit Health. 2025 Jun 9;11:20552076251349624. doi: 10.1177/20552076251349624 (PMC12159478; doi:10.1177/20552076251349624)
Supplement: sj-docx-3-dhj-10.1177_20552076251349624 - Supplemental material for Motivators and barriers to the uptake of digital health platforms for family planning services in Lagos, Nigeria: A mixed-methods study [file sj-docx-3-dhj-10.1177_20552076251349624.docx]

**A Multimethod Exploratory Study to Generate Insights for Optimizing E-pharmacy for Family Planning in Nigeria**

**Background**

The primary objective of this survey is to transform the insights generated from the qualitative study into quantifiable data. Through this survey, we aim to systematically gather information and insights that will allow us to analyze, understand, and quantify various aspects related to family planning and digital health services among the current users of online pharmacies for family planning (FP ePharmacy Users), users of online pharmacies for purposes other than family planning (non-FP users), users of physical pharmacy for family planning (Brick and mortar users), and individual that do not use family planning product and services (non-FP users).

**Data collection Approach**

Data will be collected through the use of SurveyCTO either self-administered (online) or physical Q&A administered by field data collectors.

**Target participants and sample size**

This survey will target a total of n = 1200 participants (300 in 4 categories) and prioritize women, adolescent and young girls within the ages of 18 and 24 years. We will aim to collect n+15% to mitigate low quality and incomplete responses from self-administered surveys.

| **Category** | **Target sample** | **Sampling pool** |
| --- | --- | --- |
| FP ePharmacy Users | 300 + 45 |  |
| non-FP ePharmacy Users | 300 + 45 |  |
| FP Brick-and-mortar (BnM) Pharmacy users | 300 + 45 |  |
| non-FP Users | 300 + 45 |  |

**Introduction**

The Busara Center for Behavioral Economics and the Society for Family Health (SfH), both non-profit organizations, are collaborating to explore the barriers and opportunities for improving the demand and uptake of traditional and online family planning services in Nigeria.

**Consent**

Your participation in this research is completely voluntary and requires you to provide us with informed consent as given in this section. As a part of the process of informed consent, you should be aware that:

1. Your participation is completely voluntary, and it is up to you to decide to take part or not. There would be no penalty if you do not agree to take part in this study.
2. You are free to withdraw from this study at any time without any penalty.
3. If you decide to participate, you will be asked to answer a survey, which will last approximately 45 minutes. If, at any point, you are uncomfortable with the questions asked, you are welcome to say you would prefer not to answer and we will move on to the next question.
4. All information you provide will be kept confidential and anonymous, and your personal data will not be shared with anyone outside the teams conducting this project. Responses that you provided will only be collected and analyzed in an aggregate manner and individual responses will be shared with anyone.
5. We value your personal experiences and views, so feel free and comfortable to answer the questions honestly.

Please feel free to ask me any questions about the study or the consent process or your participation.

Do you provide your consent to participate in this research? Providing consent means that you agree with the following statements:

1. I confirm that I have read and understood the information related to the above study. I have had the opportunity to consider the information, ask questions, and have had these answered satisfactorily.
2. My participation is voluntary, and I may withdraw my consent and discontinue the participation in the project at any time. My refusal to participate will not result in any penalty.

Select one: **Yes** / **No (ineligible for participation)**

**PART I: ELIGIBILITY TEST/SCREENER QUESTIONS**

| **Q** | **Question** | **Options** |
| --- | --- | --- |
| 1 | What is your age? | - Less than 18 yrs old **(eligible)**  - 18 to 45 yrs old **(eligible)**  - 46 yrs old and above **(ineligible)** |
| 2 | Do you use Family Planning (FP) products or services? | - Yes  **(Uncategorized FP User) (Skip to Q3)**  - No  **(non-FP User) (continue to Q2a)** |
| 2a | If not, what type of pharmacy do you normally use for health products and services? | - I use an ePharmacy for health products and services **(non-FP ePharma User)** **(Proceed section B)** - I use physical pharmacy and health centers for health products and services. **(non-FP BnM user) (Proceed to section D)** |
| 3 | What type of pharmacy do you normally use for health products and services, including family planning? | - I use an ePharmacy for health products and services, including family planning.   **(FP ePharma User)** **(**[**Proceed to section A**](#lu2voht3htnu)**)**   - I use physical pharmacy and health centers for health products and services, including family planning. **(FP BnM user) (**[**proceed to Section C**](#j1s063uxgvo0)**)** |

PART II: DEMOGRAPHIC INFORMATION (**for all**)

| **S/N** | **Question** | **Options** |
| --- | --- | --- |
|  | What is your gender | - Female  - Male |
| 1 | What is your current marital status? | - Single / Never married  - In a relationship  - Married  - Cohabiting  - Separated/Divorced  - Widowed |
| 2 | What is your highest level of education? | - None  - Primary  - Secondary  - Post secondary (HND/OND)  - Undergraduate  - Postgraduate |
| 3 | What is your current (Major) employment status? | - Employed  - Business / self-employed  - Unemployed  - Student |
| 4 | What is your occupation (e.g., Farmer, Teacher, etc)? |  |
| 5 | What is your approximate monthly income? (optional) | - No income  - Below N100,000  - NGN 100,000 - 200,000  - NGN 200,001 - 300,000  - More than NGN 300,000 |
| 6 | What is your ethnicity | - Hausa  - Igbo  - Yoruba  - Others… (specify) |
| 7 | What is your religion? | - Islam  - Christianity  - Traditional  - Non-religious  - Other… (specify) |

PART I: DIGITAL CONNECTIVITY (**for all**)

| **Technology uptake and experiences** | | |
| --- | --- | --- |
| 8 | Do you currently use a digital device (e.g. smartphone, computer, etc)? | - Yes (continue)  - No (move to Q15) |
| 9 | Do you have access to internet connection? | - Yes  - No  - Sometimes |
| 10 | If yes, how often do you use your digital device to access the internet? | - Every day  - Several times a week  - Once a week  - Less often |
| 11 |  | - Communication (e.g., email, social media, messaging apps)  - Entertainment (e.g., streaming video, listening to music, playing games)  - Information seeking (e.g., researching topics, reading news articles, watching educational videos)  - Shopping (e.g., buying products and services online)  - Banking and finance (e.g., checking account balances, transferring money, paying bills)  - Education and learning (e.g., taking online courses, reading e-books, watching educational videos)  - Other (please specify) |
| 12 | What is your main source of health information? | - Healthcare professionals  - Online sources/searches  - Friends and family  - Others (specify)... |
| 13 | Do you trust information from online sources? | -Yes, very much.  -Yes, somewhat.  - Absolutely not  - Not usually |
| 14 | What sources do you trust for health information? | - Healthcare professionals  - Online sources/searches  - Friends and family  - Others (specify)... |

PART IV: GENERAL AWARENESS, ATTITUDES & UPTAKE OF FAMILY PLANNING (**for all**)

| 15 | What do you understand by the term Family Planning? | - Family planning is the process of providing information and services to help people decide when and how many children to have.  - Family planning is the use of contraception to prevent unintended pregnancies.  - Family planning is the practice of deciding when and how many children to have, and taking steps to achieve those goals  - Family planning is the practice of spacing pregnancies at least 2 years apart  - Family planning is the practice of limiting the number of children a couple has to two or fewer. |
| --- | --- | --- |
| 16 | What is your main source of family planning information? | - Hospitals and healthcare centers  - Online sources  - Other(specify…) |
| 17 | Which method(s) of family planning do you know? (Select all that apply) | - Monthly Pills  - DMPA-SC (Injectable)  - DMPA-IM (Injectable)  - IUD  - Implant  - Female condom  - Male Condom  - Sterilization  - Lactation amenorrhea method (LAM)  - Cycle beads  - Emergency contraceptive method  - Others |
| 18 | Which method have you ever used among the following? (select all that apply) | - Pills  - DMPA-SC (Injectable)  - DMPA-IM (Injectable)  - IUD  - Implant  - Female condom  - Male condom  - Sterilization  - Cycle beads  - Emergency contraceptive method  - Others (specify)… |
| 19 | When was the last time you used any of the selected family planning methods? | - Less than 3 months ago  - 3-6 months ago  - More than 6 months ago |
| 20 | What made you choose the Family Planning method(s) you previously used or currently use (select all that apply)? | - Safety  - Effectiveness  - Affordable  - Accessible  - Acceptable by my religion/culture  - Few side effects  - The only method I know  - Only method available  - Don’t know  - Other (specify)... |
| 21 | Where do you access the family planning method?(select all that apply) | - Hospital/Clinic  - NGOs/Charity Drive  - Local Pharmacy Shops  - Online Pharmacy  - Other (specify)... |
| 22 | Did you experience any side effects? | - Yes (continue)  - No (end section) |
| 23 | What were the most significant side effects you experienced (mention)? | *[Write freely]* |

PART V: USER CATEGORIES

| **SECTION A: FP ePharmacy User** | | |
| --- | --- | --- |
| 24 | Mention the name(s) of the ePharmacy platform that you have used | *[Write freely]* |
| 24a | How did you become aware of the ePharmacy platform(s) that you have used | - Online search  - Referrals from friends  - Referrals from healthcare providers  - Others (specify)… |
| 25 | Are you aware of telemedicine services? | - Yes (continue)  - No (move to Q27) |
| 25a | How would you define telemedicine? | *[Write freely]* |
| 26 | Have you ever accessed telemedicine services? | - Yes (continue)  - No (skip to Q26b) |
| 26a | If yes, what are some of the challenges you have experienced when using telemedicine services, including ePharmacy? | Lack of trust in online health services  Concerns about the quality of products and services  Difficulty navigating the e-pharmacy website or app  High delivery costs  Other (please specify) |
| 26b | If no, what are the main reasons for not using telemedicine services? [select all that apply] | Perceived lack of need  easy access to health information online  convenient access to physical healthcare facilities  preference for face-to-face consultations  onboarding process difficulties  Others… |
| 27 | Have you ever used diagnostic or counseling services through ePharmacy? | Yes  No |
| 28 | How often do you use ePharmacy channels for family planning compared to traditional stores? | Much less often  Less often  About the same  More often  Much more often |
| 29 | Have you found it easier to access family planning products/services through ePharmacy channels? | Yes, much easier  Yes, somewhat easier  No, no change  No, somewhat harder  No, much harder |
| 30 | Do you trust the quality of care that is provided through the ePharmacy platform? | Yes  No |
| 31 | How satisfied were you with your family planning product purchase from an ePharmacy? | - Very satisfied  - Satisfied  - Neutral  - Disappointed  - Extremely disappointed |
| 32 | Do you have any beliefs that influence your preference for online services over physical healthcare facilities? | - Lower cost  - Easy access to doctors and pharmacists  - Convenience of access from any location  - Others (specify)… |
| 33 | Do you have any unmet information needs related to ePharmacy or telemedicine services? | *[Write freely]* |
| 34 | How comfortable are you with your current family planning methods? | Very uncomfortable  Uncomfortable  Neutral  Comfortable  Very comfortable |
| 35 | Do you think there are risks to the use of ePharmacy? | Yes (continue) No (skip to Q36) Not sure (skip to Q36) |
| 35a | If yes, which of the following risks do you associate with the use of ePharmacy? [select all that apply] | Lack of product authentication  Lack of regulation  Poor quality products  Limited patient/user information  Others, please specify… |
| 35b | How concerned are you about the potential risks of using e-Pharmacy services for family planning? | Not at all concerned  Slightly concerned  Moderately concerned  Very concerned  Extremely concerned |
| 36 | Do you feel overwhelmed by the amount of information available about family planning products and services on the ePharmacy platform? | Never  Rarely  Sometimes  Often  Very often |
| 38 | What do your friends and family think about using ePharmacy and telemedicine for family planning? | Strongly disapprove  Disapprove  Neutral  Approve  Strongly approve |
| 39 | How clear is it to you what products and services for family planning are available on ePharmacy platform and how to use them? | Very unclear  Unclear  Neutral  Clear  Very clear |
| 40 | How easy is it for you to access ePharmacy services for family planning? | Very difficult  Difficult  Neutral  Easy  Very easy |
| 41 | What challenges do you face when using digital health services (ePharmacy and telemedicine) for family planning?[select multiple] | Network issues  Language Support  Delivery delays  Lack of prescriptions  Poor Pharmacist Support  Other |
| 42 | What challenges have you faced when using digital health services (ePharmacy and Telemedicine) platforms for family planning purposes, specifically related to user interface?[select multiple] | Product search issues  Unresponsive search filters  Unavailable products  Long waiting times to speak to an ePharmacist.  Can’t find the feature to speak to ePharmacists or doctors.  Other (specify)... |
| 44 | What challenges have you faced when processing payments for products or services on digital health (ePharmacy and Telemedicine) platforms? [select multiple] | Payment failures  Lack of payment method guidance  High delivery charges  Other (specify)... |
| 44 | What is you most preferred means of obtaining family planning products from an ePharmacy | Delivery (continue)  Pick up (skip to 44c) |
| 44a | If you prefer delivery, do you find the **delivery fee** for the family planning product purchased from an ePharmacy affordable? | Yes  No |
| 44b | Are you able to precisely and conveniently set your home address/delivery location for the family planning product purchased? | Yes  No |
| 44c | If you prefer pick up, do you find the store locations convenient and accessible? | Yes  No |
| 45 | Do you have adequate knowledge to browse the website/app, select family planning products or services, and complete the checkout process? | Yes  No |
| 46 | What factors influenced your decision to use an online digital health platform to purchase family planning products? | Convenience  Privacy  Cost  Selection  Other (specify)... |
| 47 a-e | How interested are you in the following enhancements to online pharmacy services? | |
|  | Reduced delivery fees? | - Not at all interested  - Slightly interested  - Moderately interested  - Very interested  - Extremely interested |
|  | Same-day delivery? | - Not at all interested  - Slightly interested  - Moderately interested  - Very interested  - Extremely interested |
|  | More Pick-Up Options? | - Not at all interested  - Slightly interested  - Moderately interested  - Very interested  - Extremely interested |
|  | Delivery Tracking Services? | - Not at all interested  - Slightly interested  - Moderately interested  - Very interested  - Extremely interested |
|  | Better Pharmacist support and Prescription guides? | - Not at all interested  - Slightly interested  - Moderately interested  - Very interested  - Extremely interested |
|  | Wider product selection? | - Not at all interested  - Slightly interested  - Moderately interested  - Very interested  - Extremely interested |
|  | Better customer service? | - Not at all interested  - Slightly interested  - Moderately interested  - Very interested  - Extremely interested |
|  | Educational resources? | - Not at all interested  - Slightly interested  - Moderately interested  - Very interested  - Extremely interested |
|  |  |  |
| **SECTION B: non-FP ePharmacy User** | | |
| 24 | Mention the name(s) of the ePharmacy platform that you have used | *[Write freely]* |
| 24a | How did you become aware of the ePharmacy platform(s) that you have used | - Online search  - Referrals from friends  - Referrals from healthcare providers  - others… |
| 25 | What are the main reasons why you have not used ePharmacies for family planning? | Lack of trust in online pharmacies  Concerns about the quality of products and services  Difficulty navigating the e-pharmacy website or app  High shipping costs  Other (please specify) |
| 26 | Are you aware of telemedicine services? | Yes (continue)  No (skip to Q28) |
| 27 | How would you define telemedicine? |  |
| 28 | Have you ever accessed telemedicine services? | - Yes (continue)  - No (skip to Q28b) |
| 28a | If yes, what are some of the challenges you have experienced when using telemedicine and ePharmacy services? | Lack of trust in online health services  Concerns about the quality of products and services  Difficulty navigating the ePharmacy website or app  High delivery costs  Other (specify)... |
| 28b | If no, what are the main reasons for not using telemedicine services? [select all that apply] | Perceived lack of need  easy access to health information online  convenient access to physical healthcare facilities  preference for face-to-face consultations  onboarding process difficulties  Other (specify)... |
| 29 | If you were to order family planning products from an ePharmacy, would you be able to afford the delivery fee? | Yes  No |
| 30 | Are you worried about your personal information being kept confidential when you use digital Health (ePharmacy & telemedicine) platforms for family planning? | Yes  No |
| 31 | Do you think there are risks to the use of ePharmacy? | Yes (continue) No (skip to Q32) Not sure (skip to Q32) |
| 31a | If yes, which of the following risks do you associate with the use of ePharmacy? [select all that apply] | Lack of product authentication  Lack of regulation  Poor quality products  Limited patient/user information  Others, please specify… |
| 31b | How concerned are you about the potential risks of using e-Pharmacy services for family planning? | Not at all concerned  Slightly concerned  Moderately concerned  Very concerned  Extremely concerned |
| 32 | What do your friends and family think about using ePharmacy and telemedicine for family planning? | Strongly disapprove  Disapprove  Neutral  Approve  Strongly approve |
| 33 | What challenges have you faced when using digital health services (ePharmacy and Telemedicine) platforms, specifically related to user interface?[select multiple] | Product search issues  Unresponsive search filters  Unavailable products  Long waiting times to speak to an ePharmacist.  Can’t find the feature to speak to ePharmacists or doctors.  Other (specify)... |
| 34a-e | How interested are you in the following enhancements to online pharmacy services? |  |
|  | Reduced delivery fees? | - Not at all interested  - Slightly interested  - Moderately interested  - Very interested  - Extremely interested |
|  | Same-day delivery? | - Not at all interested  - Slightly interested  - Moderately interested  - Very interested  - Extremely interested |
|  |  |  |
|  |  |  |
|  |  |  |
|  | Wider product selection? | - Not at all interested  - Slightly interested  - Moderately interested  - Very interested  - Extremely interested |
|  | Better customer service? | - Not at all interested  - Slightly interested  - Moderately interested  - Very interested  - Extremely interested |
|  | Educational resources? | - Not at all interested  - Slightly interested  - Moderately interested  - Very interested  - Extremely interested |
|  |  |  |
| **SECTION C: BnM Pharmacy User** | | |
| 24 | Are you aware of ePharmacy? | Yes  No |
| 25 | What are the main reasons why you have not used e-pharmacies for family planning?[select multiple] | Lack of trust in online pharmacies  Concerns about the quality of products and services  Difficulty navigating the e-pharmacy website or app  High shipping costs  Other (please specify) |
| 26 | Are you aware of telemedicine services? | Yes  No |
| 27 | How would you define telemedicine? |  |
| 28 | What are the main reasons for not using telemedicine services?[select multiple] | Perceived lack of need  easy access to health information online  convenient access to physical healthcare facilities  preference for face-to-face consultations  onboarding process difficulties |
| 29 | What factors would make you more likely to use telemedicine services? |  |
| 30 | What are the potential challenges you anticipate when using digital health (ePhamarcy & telemedicine) platforms for family planning products and services? | Lack of trust in online pharmacies  Concerns about the quality of products and services  Difficulty navigating the epharmacy website or app  High delivery costs  Other (please specify) |
| 31 | If you were to order family planning products from an ePharmacy, would you be able to afford the delivery fee? | Yes  No |
| 32 | Are you worried about your personal information being kept confidential when you use digital Health platforms? | Yes  No |
| 33 | Do you think there are risks to the use of digital health (ePharmacy and telemedicine) platforms? | Yes (continue) No (skip to Q34) Not sure (skip to Q34) |
| 33a | If yes, which of the following risks do you associate with the use of ePharmacy? [select all that apply] | Lack of product authentication  Lack of regulation  Poor quality products  Limited patient/user information  Others, please specify… |
| 33b | How concerned are you about the potential risks of using e-Pharmacy services for family planning? | Not at all concerned  Slightly concerned  Moderately concerned  Very concerned  Extremely concerned |
| 34 | What do your friends and family think about using digital health services (ePharmacy and telemedicine) for family planning? | Strongly disapprove  Disapprove  Neutral  Approve  Strongly approve |
| 35 | Do you have any unmet information needs related to ePharmacy or telemedicine services? |  |
| 36 | How much do you trust the recommendations of health care professionals about digital health (ePharmacy and telemedicine) services for family planning? | Not at all  A little bit  Moderately  Quite a lot  Completely |
| 37 | Are there any beliefs or concerns in your community that hinder the use of digital health (ePharmacy and telemedicine) services? | specify… |
|  |  |  |
| **SECTION D: non-FP User** | | |
| 24 | How often do you visit a physical pharmacy for medications? | - Once a year or more  - Every few years  - Once or twice in my lifetime  - Never |
| 25 | How often do you visit a healthcare provider for general checkups or consultations? | - Once a year or more  - Every few years  - Once or twice in my lifetime  - Never |
| 26 | What type of pharmacy do you typically visit for your medications? | - Community Pharmacies (located around residential areas)  - Hospital Pharmacies (located within hospitals and other medical facilities)  - Other (please specify) |
| 27 | What type of healthcare provider do you typically see? | - General practitioner  - Obstetrician-gynecologist  - Other (please specify) |
| 28 | Have you ever discussed family planning with a pharmacist or healthcare provider? | - Yes  - No |
| 29 | Do you believe that family planning is an important topic for individuals and couples? Why or why not? | - Yes, I believe that it is important for individuals and couples to be able to make informed decisions about their family size and spacing.  - No, I do not believe that family planning is an important topic.  - I am not sure whether or not family planning is important. |
| 30 | What are your thoughts on the use of modern family planning methods? | - I believe that modern family planning methods are safe and effective.  - I have some concerns about the use of modern family planning methods.  - I am not sure what to think about modern family planning methods. |
| 31 | Do you have any concerns about using family planning methods? | - Yes, I have concerns about the side effects of family planning methods.  - Yes, I have concerns about the effectiveness of family planning methods.  - I am concerned about the difficulty of obtaining and using family planning methods  - No, I do not have any concerns about using family planning methods. |
| 32 | Are there any religious or cultural beliefs in your community that influence your attitudes towards family planning? | - Yes, there are religious or cultural beliefs in my community that influence my attitudes towards family planning.  - No, there are no religious or cultural beliefs in my community that influence my attitudes towards family planning.  - I am not sure whether or not there are religious or cultural beliefs in my community that influence my attitudes towards family planning. |
| 33 | Are you aware of e-pharmacy? | - Yes (continue)  - No (skip to 35) |
| 34 | What are the main reasons why you have not used ePharmacies for any healthcare services/products? | - Lack of trust in online pharmacies  - Concerns about the quality of products and services  - Difficulty navigating the ePharmacy website or app  - High shipping costs  - Other (please specify) |
| 35 | Are you aware of telemedicine services? | - Yes (continue)  - No (skip to 39) |
| 36 | How would you define telemedicine? | - |
| 38 | What are the main reasons for not using telemedicine services? | - Perceived lack of need  - easy access to health information online  - convenient access to physical healthcare facilities  - preference for face-to-face consultations  - onboarding process difficulties  -Other(Specify) |
| 39 | What factors would make you more likely to use digital health service (ePharmacies and telemedicine) platforms? | - |
| 40 | What are the potential challenges you anticipate when using digital health (ePharmacy & telemedicine) for both family planning and any other healthcare needs?[select multiple] | - Lack of trust in online pharmacies  - Concerns about the quality of products and services  - Difficulty navigating the e-pharmacy website or app  - High delivery costs  - Other (please specify) |
| 41 | What factors would influence your decision to use an online digital health platform to purchase family planning products and other healthcare services/products?[select multiple] | - Convenience  - Affordability  - Delivery options  - Customer service  - Product Selection  - Educational resources  - Brand reputation  - Other (Please specify) |

PART VI: WILLINGNESS TO PAY FOR FP PRODUCTS & SERVICES

1. Imagine that you are considering purchasing a new type of family planning product that is more effective than the one you are currently using. How much more would you be willing to pay for this new product?

a. between 1% and 10% more

b. between 11% and 20% more

c. between 21% and 30% more

d. between 31% and 40% more

d. Greater than 40% more

e. Not willing to pay more

f. Don’t know

2. How much less would you be willing to pay for the family planning products you are purchasing if you were concerned about the privacy of your purchase?

- between 1% and 10% less
- between 11% and 20% less
- between 21% and 30% less
- between 31% and 40% less
- greater than 40% less
- not willing to pay less
- don’t know

3. Imagine that you are considering purchasing a family planning product that is more expensive than the product you are currently using, but it is also more convenient to purchase. How much more would you be willing to pay for this more convenient product?

- between 1% and 10% less
- between 11% and 20% less
- between 21% and 30% less
- between 31% and 40% less
- greater than 40% less
- not willing to pay less
- don’t know

4. How comfortable would you be using online consultation services with a family planning specialist?

- Very comfortable
- Comfortable
- Neutral
- Uncomfortable
- Very uncomfortable
- Don’t know

5. How much would you be willing to pay for a basic virtual consultation with a family planning specialist that includes ONLY prescription of appropriate family planning products?

- Less than N3000
- N3000-N5000
- N5001- N7000
- N7001 – N9000
- More than N9000

6. How much more would you be willing to pay for an in-depth virtual consultation with a family planning specialist that includes personalized recommendations and advice, compared to a basic virtual consultation?

- between 1% and 10% less
- between 11% and 20% less
- between 21% and 30% less
- between 31% and 40% less
- greater than 40% less
- not willing to pay less
- don’t know

7. How much more would you be willing to pay for a virtual consultation package that includes follow-up sessions and ongoing support from a family planning specialist, compared to a basic virtual consultation?

- between 1% and 10% less
- between 11% and 20% less
- between 21% and 30% less
- between 31% and 40% less
- greater than 40% less
- not willing to pay less
- don’t know

8. How likely are you to consider using virtual consultation services with a family planning specialist if they were priced within your preferred price range?

- Very likely
- Likely
- Neutral
- Unlikely
- Very unlikely
